# The evolution of conglobation in Ceratocanthinae

**DOI:** 10.1038/s42003-022-03685-2

**Published:** 2022-08-06

**Authors:** Yuanyuan Lu, Alberto Ballerio, Shuo Wang, Zhengting Zou, Stanislav N. Gorb, Tao Wang, Lulu Li, Shen Ji, Zhengyu Zhao, Sheng Li, Yijie Tong, Yandong Chen, De Zhuo, Cihang Luo, Weiwei Zhang, Ning Liu, Qi Gu, Ming Bai

**Affiliations:** 1 Key Laboratory of Zoological Systematics and Evolution, Institute of Zoology, Chinese Academy of Sciences, Beijing, China; 2 Viale Venezia 45, Brescia, Italy; 3 State Key Laboratory of Membrane Biology, Institute of Zoology, Chinese Academy of Sciences, Beijing, China; 4 Beijing Institute for Stem Cell and Regenerative Medicine, Beijing, China; 5 University of Chinese Academy of Sciences, Beijing, China; 6 Department of Functional Morphology and Biomechanics, Zoological Institute, University of Kiel, Kiel, Germany; 7 School of Agriculture, Ningxia University, Yinchuan, China; 8 College of Plant Protection, Hebei Agricultural University, Baoding, 071001 China; 9 Beijing Xiachong Amber Museum, Beijing, China; 10 State Key Laboratory of Palaeobiology and Stratigraphy, Nanjing Institute of Geology and Palaeontology, Chinese Academy of Sciences, Nanjing, China; 11 P.O. Box 4680, Chongqing, China; 12 Hainan Yazhou Bay Seed Lab, Building 1, No.7 Yiju Road, Yazhou District, Sanya City, Hainan 572025 China

**Keywords:** Entomology, Animal behaviour

## Abstract

Conglobation is an adaptive behaviour occurring independently in various animal groups. Here, we study the evolution of conglobation in Ceratocanthinae, a beetle group with the ability to roll three body segments into a tight ball. It is here implied that this ability evolved only once in the Mesozoic. Evidence is offered suggesting that the high defensive strength of Ceratocanthinae is due not only to the spherical body shape but also to the thickness and stronger mechanical properties of the dorsal cuticle. We further validate five adaptive characters including the allometrically thickened body wall and find that the specific adaptation of different body segments are likely separate evolutionary events. Finally, we propose an “attackers stress” hypothesis to explain the origin of conglobation behaviours. This work contributes to understanding how and why conglobation behaviour may have evolved in this group.

## Abstract (web summary)

Evolutionary and functional analyses alongside new fossil specimens expand our understanding of rolling-into-ball behaviour in pill scarab beetles.

## Introduction

**Fig. 1:**
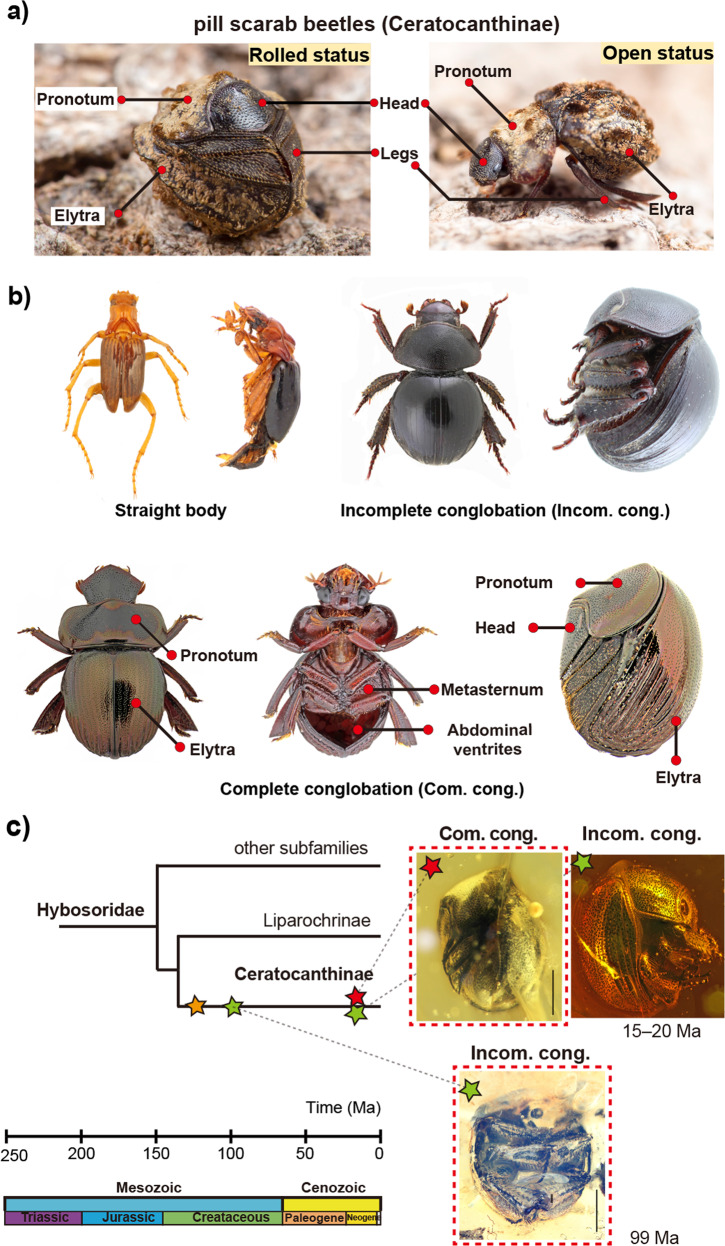
Conglobation behaviours of pill scarab beetles. **a** Two postures of a pill scarab beetle with complete conglobation: *Paulianostes panggoling*, Malaysia (Insecta: Coleoptera: Ceratocanthinae). **b** Three archetypes of conglobation in Ceratocanthinae: straight body (*Ivieolus brooksi*), incomplete conglobation (*Acanthocerodes* sp.), and complete conglobation (*Synarmostes tibialis*). **c** Fossil records of Ceratocanthinae. Yellow stars indicate fossils with a straight-body archetype from the Yixian Formation (125 Ma) (*Mesoceratocanthus tuberculifrons*), green stars indicate fossils with an incomplete conglobation archetype from Kachin amber (99 Ma) (*Palaeopycnus circus* Lu, Ballerio & Bai sp. nov.) and from Dominican amber (15–20 Ma) (*Germarostes* sp.), and red stars indicate fossils with a complete conglobation archetype from Dominican amber (15–20 Ma) (*Nesopalla succini* Lu, Ballerio & Bai sp. nov.). Red dashed box indicates new reports in here. a ©Zhengzhong Huang.

Some animals can curl their body into a ball (i.e., conglobation behaviours), an ability that is particularly well known in mammals such as armadillos, pangolins and hedgehogs^1–3^, as well as in some arthropods^4–6^. For example, pill millipedes (Glomerida and Sphaerotheriida)^4^ and pill woodlice (Armadillidiidae)^5^ (Supplementary Fig. 1a, b) are able to roll all of their body segments to form a tight ball (i.e., complete conglobation). Conversely, due to the presence of only three body segments, the majority of insect species displaying curling behaviour can merely form a loose ball (i.e., incomplete conglobation). Such insects include Chrysididae (Hymenoptera), Leiodidae, Cybocephalidae, Clambidae (Coleoptera) and some Blaberidae (Blattoidea)^7–11^ (Supplementary Fig. 1c, d). Within beetles, Ceratocanthinae (pill scarab beetles) are a morphologically unique group able to form a tight ball through a process that involves not only curling the three body trunk segments but also folding the tibiae (Fig. 1a), a feature that astounded Darwin, who, during the “Beagle” expedition, described the behaviour as “rolls up like Armadillo”^12^.

Ceratocanthinae are a subfamily of Hybosoridae (Coleoptera, Scarabaeoidea) and currently include ~449 species described in 44 genera^13,14^. They are mainly distributed in pantropical regions, and adults are often found in termite nests/leaf litter/rotten wood, where they are hypothesized to feed on fungi or debris^13^. In terms of conglobation, they display three archetypes: straight body, incomplete conglobation and complete conglobation^12,15^ (Fig. 1b, Supplementary Table 1). The most up-to-date phylogenetic analysis supports the hypothesis that conglobation capacity evolved in Ceratocanthinae only once^13,16^. To date, only two extinct species are known in this subfamily: one straight-bodied Mesozoic species without conglobation ability (whose attribution to Ceratocanthinae is doubtful)^17^ and one Cenozoic species with incomplete conglobation^18,19^ (Fig. 1c). The early history and evolution of this group are thus poorly known.

A few studies on functional morphology in arthropods hypothesized that conglobation behaviours have defensive^20,21^ and/or physiological functions (for example, moisture retention and thermoregulation)^13,22^. For Ceratocanthinae, the capability of rolling the body into a ball might have evolved for higher defensive strength to support a lifestyle in the hostile environment of termite nest^13^. Our knowledge about the conglobation behaviour, however, is limited to a few scattered reports^8,13,23,24^.

In this paper, by using integrated methodology, we investigate the questions of how and why the unique conglobation behaviour has evolved in pill scarab beetles. Firstly, we describe one new extinct genus and four new extinct species, which represent the earliest examples of incomplete and complete conglobation within scarab beetles and, more generally, within beetles^25^. Second, from the functional morphology and mechanical engineering points of view, we verify the higher defensive strength of Ceratocanthinae when compared with that of the other species of Scarabaeoidea. Third, by combining knowledge from intensive studies of both extinct and extant species, we validate five adaptive characters and explore the evolutionary history of three body trunk segments. Finally, we propose an “attackers stress” hypothesis in order to explain the origin of conglobation behaviours.

## Results and discussion

### Systematic palaeontology

Order Coleoptera Linnaeus, 1758^26^

Family Hybosoridae Erichson, 1847^27^

Subfamily Ceratocanthinae Martínez, 1968^28^

### Genus: *Palaeopycnus* Lu, Ballerio & Bai gen. nov

LSID: urn:lsid:zoobank.org:act:FC9EFB6C-5345-441B-860B-CBB369B59685

#### Type species

*Palaeopycnus circus* Lu, Ballerio & Bai sp. nov.

#### Etymology

From Ancient Greek “Palaeos” (=ancient) and the Latinized word “Pycnus” from Ancient Greek “Pycnos” (=compact). The gender is masculine.

#### Diagnosis

A small Scarabaeoidea with labrum exposed and not aligned to the longitudinal axis of clypeus, mandibles not protruding and not visible from above, genal canthus present and more or less at a right angle with respect to clypeus, antennae with club made of three antennomeres, clypeus subrectangular, pronotum and head capable of being bent inwards to form a loose ball, scutellum triangular wider than long, elytra strongly convex, meso- and metatibiae lacking transverse carinae and with multiple sides.

#### Remarks

*Palaeopycnus* gen. nov. is here tentatively assigned to the subfamily Ceratocanthinae mainly due to the combination of the head and pronotum capable of being bent inwards, labrum not aligned with the longitudinal axis of the clypeus, lack of protruding mandibles, presence of a genal canthus and shape of the meso- and metatibiae as well as because of the lateroventral expansion of the prothoracic hypomeron and the sides of the distal portion of exposed scutellum being weakly concave and forming an acutely pointed apex. It must be stressed, however, that because other key characters highlighted by Ballerio & Grebennikov^13^, i.e., prosternal aphophyses of the prothorax reaching the inner wall of the pronotum and posterior projection beyond the posterior metatergite edge on the metascutal furrow as well as wing characters, were not visible, the assignment to Ceratocanthinae remains tentative. The addition of the few available characters to the matrix revised from Ballerio & Grebennikov (Supplementary Note 1, Supplementary Data 1, Supplementary Fig. 2) resulted in the placement of the genus *Palaeopycnus* within the Ceratocanthinae as a basal genus of a clade comprising all Ceratocanthini and excluding Ivieolini and Scarabatermitini. The shortage of available characters could, however, generate artefacts; therefore, our results must be interpreted cautiously. It is therefore also possible that *Palaeopycnus* could represent an extinct lineage within Scarabaeoidea worth of a separate family status. Finally, we provide some notes on the remaining subfamilies of Hybosoridae and the reasons why we exclude the possibility of *Palaeopycnus* belonging to any of them: the Hybosorinae are unable to bend the head and pronotum and are characterized by the presence of a cupuliform first antennomere of the antennal club (the only antennal club image available for *Palaeopycnus* does not reveal the presence such a convex antennomere) and the presence of protruding mandibles that are clearly visible. The Anaidinae are unable to bend the head and pronotum and have protruding mandibles, too. Finally, the Liparochrinae, which are the subfamily displaying the strongest resemblance to *Palaeopycnus*, despite being able to bend the head and prothorax, have protruding mandibles. Therefore, among the Hybosoridae subfamilies, the only one with characters that match those visible in the available specimens of *Palaeopycnus* is Ceratocanthinae.

**Fig. 2:**
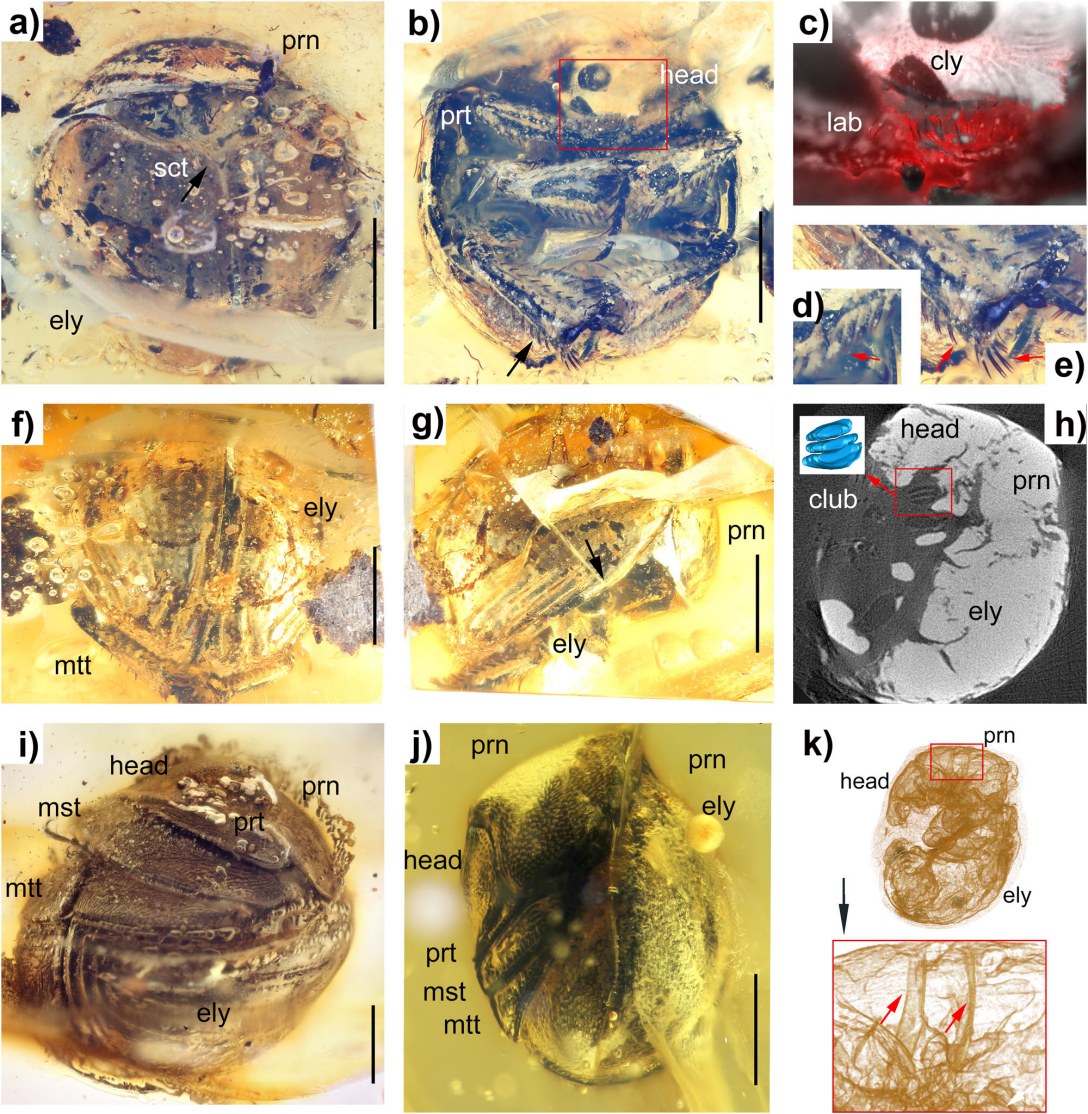
Images of Mesozoic and Ceonzoic amber of Ceratocanthinae. **a**–**h** *Palaeopycnus circus* Lu, Ballerio & Bai sp. nov. (holotype). **a** General habitus, dorsal view. **b** General habitus, ventral view, with details of the rectangular frame shown in **c**. The black arrow points to bifid setae on the tibia (details in **d** and **e**). **c** Head, showing the clypeus and labrum under red epifluorescence. **d** Left mesotibia; the red arrow points to bifid setae on the posterior part. **e** Right metatibia; red arrows point to bifid setae on the posterior and terminal parts. **f** Posterior part of the dorsal view. **g** General habitus, lateral view. The black arrow points to the marginal area of the elytron. **h** Microtomographic slide showing an antenna in lateral view, where the red rectangle shows the lamellate club of the antenna. The screenshot image from the 3D surface model of the club is shown in the white rectangle. **i** *Ceratocanthus huarongii* Lu, Ballerio & Bai sp. nov. (holotype). General habitus, lateral view. **j**, **k** *Nesopalla succini* Lu, Ballerio & Bai sp. nov. (holotype). **j** General habitus, lateral view. **k** Lateral view, where the red rectangle and arrows show prosternal apophyses that reach the inner wall of the pronotum. Scale bars: 1 mm. Abbreviations: prn, pronotum; ely, elytron; sct, scutellum; prt, protibia; cly, clypeus; lab, labrum; mad, mandible; mst, mesotibia; mtt, metatibia.

### *Palaeopycnus circus* Lu, Ballerio & Bai sp. nov. (Fig. 2a–h, Supplementary Fig. 3)

LSID: urn:lsid:zoobank.org:act:277AC424-C435-4524-A2D4-864920BDF2F5

#### Etymology

Noun in apposition. The specific name is derived from Latin adjective circus (= rounded), referring to its conglobation body shape.

#### Material

Holotype in Kachin amber from the Hukawng Valley (Myanmar), gender unknown. The type specimen (NIGP180664), is deposited in the Nanjing Institute of Geology and Palaeontology, Chinese Academy of Sciences, Nanjing, Jiangsu, China (NIGPAS).

#### Description. Holotype

Length 2.3 mm (from base of pronotum to the end of the elytra); width 1.8 mm. Small-sized Ceratocanthinae, with incomplete “rolling up” coaptations. Head and pronotum deflexed, forming a loose subsphaerical ball. **Body Colour:** Head, pronotum, elytra, and legs uniformly dark brown (colour probably altered by fossilization). Glabrous (apart from legs). **Head:** wide (W/L ratio = 1.4 (0.7 mm/0.5 mm)), wider than long, fore portion almost semicircular (covered with some white impurities), not reflexed upward; head dorsal surface without distinct impressed punctures, labrum not aligned to the axis of clypeus, with sparse erect setation. Antennal club made of three antennomeres. **Pronotum:** subtrapezoidal, wider than long (W/L ratio = 1.4 (1.5 mm/1.1 mm)), narrower than elytra; fore margin feebly bisinuate; fore angles triangular, acutely pointed; fore edge continuously margined, edges of sides finely margined (dorsal view), base continuously strongly margined; base at middle not protruding backwards; pronotal surface regularly convex without paradiscal depressions. Shiny, smooth, without distinct punctures. **Scutellum:** about as wide as long, sides convergent to form a triangle with acute apex. Without distinct punctures. **Elytra:** wider than long (W/L ratio = 1.1 (1.8 mm/1.6 mm)), apical fourth regularly rounded (dorsal view), with one irregular protuberance near the end of each elytron; elytra regularly convex; elytral sutural stria distinct and feebly raised on median and apical third; elytra with several longitudinal striae of large simple punctures (interpunctural distance about twice the diameter of punctures) plus at least three longitudinal furrows on elytral sides on median and apical thirds. **Legs:** protibiae straight, with at least one longitudinal carina and several large sharp setae all over its length, outer margin with at least five irregular blunt teeth, the two distal ones are more developed. Mesotibiae relatively thick and strong, subtriangular with at least two longitudinal carinae on outer face, each carina bearing a row of large sharp apically bifid setae, apex with at least two large apical spurs and at least four short blunt strong setae, mesotarsi with five tarsomeres, first tarsomere longer than second tarsomere, second tarsomere about the same size of third tarsomere, third tarsomere about as long as fourth tarsomere, fifth tarsomere longer and thinner than fourth tarsomere, apical claws small and feebly curved, each tarsomere bearing basally some short sharp setae. Metatibiae relatively thick and strong, subtriangular, with at least four longitudinal carinae visible on outer face, each carina bearing a row of large sharp strong apically bifid setae, apical portion of metatibia ending with two straight apical strong spurs and some long strong sharp apically bifid setae.

#### Diagnosis

*P*. *circus* sp. nov. differs from *P*. *fushengii* sp. nov. the only other known species of the genus, because of the following combination of characters: (a) elytral surface regular and smooth (apart from the rows of punctures and the longitudinal furrows), (b) elytral apex normal, without a protruding cariniform process, (c) pronotum smooth with regular surface, (d) head smooth with regular surface, and (e) meso- and metatibiae with setae sharp and apically bifid.

#### Remarks

We examined a second specimen (NIGP180665, supplementary Supplementary Fig. 3), tentatively attributed here to *P*. *circus* sp. nov., which is so heavily damaged and badly preserved that its observation is particularly difficult. It has the same elytral sculpturing as the holotype but differs from it in the setation of the meso- and metatibiae not being apically bifid; furthermore, the clypeus is subrectangular.

### *Palaeopycnus fushengii* Lu, Ballerio & Bai sp. nov. (Supplementary Fig. 4a–g)

LSID: urn:lsid:zoobank.org:act:E439A7D8-5125-459F-A6C6-7456F2989552

#### Etymology

Noun in the genitive case. The species is named in honour of outstanding Chinese entomologist Fusheng Huang, who passed away in 2021. At the same time, the name Fusheng in Chinese language has the meaning of “resurgent”, which evocates the characteristic of insects preserved in amber which look like if they were still alive.

#### Material

Holotype in Kachin amber, gender unknown. The type specimen (No. BXAM BA-COL-001), housed in the Institute of Zoology, Chinese Academy of Sciences (IZAS), Beijing, China, will be eventually deposited in the Beijing Xiachong Amber Museum, Beijing, China. Specimen is available for study by contacting MB or DZ.

#### Description: Holotype

Length 2.8 mm (from base of pronotum to the end of the elytra); width 2.5 mm. Small-sized Ceratocanthinae, with incomplete “rolling up” coaptations. Head and pronotum deflexed, forming a subsphaerical ball. **Body Colour:** Head, pronotum, elytra, and legs dark brown (colour probably altered by fossilization). Glabrous (apart from legs). **Head:** wide (W/L ratio = 1.2 (0.8 mm/0.65 mm)), oval, fore portion irregularly semicircular (covered with some white impurities), not reflexed upward; genal canthus present and more or less at a right angle with respect to clypeus, genal canthus limited to bordering the fore portion of eyes, head dorsal surface irregular, with distinct impressed punctures, labrum not aligned to the axis of clypeus, with sparse erect setation. **Pronotum:** subtrapezoidal, wider than long, narrower than elytra fore margin feebly bisinuate; fore angles triangular, acutely pointed; fore edge continuously margined, edges of sides with finely margined (dorsal view), base continuously strongly margined; base at middle not protruding backwards; pronotal surface irregularly convex with paradiscal depressions, pronotal surface with distinct punctures. **Scutellum:** about as wide as long, sides convergent to form a triangle with acute apex. **Elytra:** wider than long (W/L ratio = 1.1 (1.8 mm/1.6 mm)), apical fourth regularly rounded (dorsal view), with one irregular protruding carina near the end of each elytron; humeral callus distinctly visible, elytra regularly convex; elytral sutural stria distinct and feebly raised on median and apical third; elytra with several longitudinal striae made of irregular punctures, at least some of them transversely comma-shaped. **Legs:** protibiae straight, with several large sharp setae all over its length, outer margin distally with at least two outer teeth. Mesotibiae relatively thick and strong, subtriangular with rows of large sharp apically pointed setae, mesotarsi with five tarsomeres, first tarsomere longer than second tarsomere, second tarsomere about the same size of third tarsomere, third tarsomere about as long as fourth tarsomere, fifth tarsomere longer and thinner than fourth tarsomere, apical claws long and feebly curved, each tarsomere bearing basally some short sharp setae. Metatibiae relatively thick and strong, subtriangular, bearing rows of large sharp strong apically pointed setae. Metatarsi, tarsomeres short and plump, from the first to fifth, each one bearing a tuft of relatively long setae basally, gradually becoming shorter, last tarsomere with two claws large and feebly curved.

#### Diagnosis

*P*. *fushengii* sp. nov. differs from *P*. *circus* sp. nov. because of the following combination of characters: (a) elytral surface irregular with several depressions, (b) elytral apex with a protruding cariniform process, (c) pronotum with irregular surface and several impressed irregular punctures, (d) head with irregular surface and strong punctation, and (e) meso- and metatibiae with setae sharp and normally pointed.

Tribe Ceratocanthini Martínez, 1968^28^

### *Ceratocanthus huarongii* Lu, Ballerio & Bai sp. nov. (Fig. 2i, Supplementary Fig. 5a–h)

LSID: urn:lsid:zoobank.org:act:00AC0E49-1ED8-4C3E-BB10-DF6FCE964D9C

#### Etymology

Noun in the genitive case. Named after Mr. Huarong Chen, who kindly donated the specimen to the Chinese Academy of Sciences.

#### Material

Holotype in Dominican amber, probably a female. The type specimen No. 2016-H-Col-015 housed in the Institute of Zoology, Chinese Academy of Sciences (IZAS), Beijing, China. Specimen is available for study by contacting MB or HRC.

#### Description. Holotype

Length 4.5 mm (from base of pronotum to the end of the elytron); width 3.9 mm. Medium-sized *Ceratocanthus*. Punctures and striae not clearly visible, due to impurities, although the elytra bear some longitudinal furrows on apical third, the striae in legs are clearly visible. **Body Colour:** head, pronotum, elytra, and legs dark brown (possibly discoloured by time). Glabrous. **Head:** wide (W/L ratio = 1.7 (2.6 mm/1.5 mm)), subpentagonal, clypeus triangular, apex forming an obtuse angle (about 130°), both sides of clypeus rectilinear, not reflexed upward, tip of clypeus rounded; genal canthus present, dorsal ocular area large, dorsal interocular area about six times the maximum width of the dorsal ocular area; head dorsal surface without distinct impressed punctures. Antennae with at least nine antennomeres visible (micro-CT scanning image without a clear resolution). **Pronotum:** subtrapezoidal, wider than long (W/L ratio = 1.9 (3.9 mm/2.1 mm)), wider than elytra; fore margin feebly bisinuate; fore angles triangular, acutely pointed; fore edge continuously finely margined, edges of sides with finely margined (dorsal view), base continuously strongly margined; base at middle not protruding backwards; pronotal surface regularly convex without paradiscal depressions. Shiny, smooth, without distinct punctures. **Scutellum:** about as wide as long (W/L ratio = 1.7 (2.0 mm/1.2 mm)), sides convergent to form a triangle with unclear likely rounded apex. Without distinct punctures. **Elytra:** wider than long (W/L ratio = 1.3 (5.4 mm/4.2 mm)), apical fourth regularly rounded (dorsal view), apex slightly re-entering inward (lateral view); elytra regularly convex; elytral suture finely raised; elytra with at least seven longitudinal rows of close small simple punctures starting from elytral sides and occupying at least the median third of each elytron. Elytral apical third with at least five deep longitudinal furrows, area between furrows distinctly raised. **Legs:** protibiae straight, outer margin with denticles, near apex with three teeth, triangle; mesotibiae and metatibiae with outer face covered by transverse shallow dense lines. Metatibiae showed a groove along the inner side where the tarsi were concealed once the beetle conglobates (micro-CT images).

#### Diagnosis

A *Ceratocanthus* characterized by the combination of large dorsal ocular area, presence of longitudinal rows of close simple punctures on elytra, raised apical elytral carinae and transverse shallow dense lines on outer face of metatibiae.

#### Remarks

*Ceratocanthus huarongii* sp. nov., because of the combination of a large dorsal ocular area and the presence of longitudinal rows of close simple punctures on the elytra, can be compared only to the following four extant species: *Ceratocantus aeneus* MacLeay, 1819^29^, *C. pecki* Paulian, 1982^30^, *C. steinbachi* Paulian, 1982^30^, and *C. clypealis* (Lansberge, 1887)^31^. It differs from *C. aeneus* because the latter has a smaller dorsal ocular area and sparser transverse lines on the metatibia outer face. It differs from *C. steinbachi*, *C. pecki* and *C. clypealis* because these three species lack longitudinal rows of punctures on the elytral sides and the remaining rows are made of punctures greatly distanced from each other (interpunctural distance more than twice the diameter of punctures), whereas in *C. huarongii* sp. nov., the punctures are very close to each other (almost touching each other). This is the first fossil record for the genus *Ceratocanthus*.

### *Nesopalla succini* Lu, Ballerio & Bai sp. nov. (Fig. 2j–k, Supplementary Fig. 5i–o)

LSID: urn:lsid:zoobank.org:act:82C7EFA6-F512-4686-8FDE-319136F3EBB7

#### Etymology

Genitive case of the Latin noun succinum (=amber).

#### Material

Holotype in Dominican amber, gender unknown. The type specimen (No. 001493), currently housed in the Institute of Zoology, Chinese Academy of Sciences (IZAS), Beijing, China, will be eventually deposited in the Three Gorges Entomological Museum, Chongqing, China. Specimen is available for study by contacting MB or WWZ. This is the same specimen illustrated in the book Frozen Dimensions^32^.

#### Description. Holotype

Length 3.2 mm (rolled up); width 2.6 mm (the maximum width of pronotum). Small-sized Ceratocanthinae, “rolling up” coaptations perfect. **Body Colour:** head, pronotum, elytra, and legs dark brown. Glabrous. Head: wide (W/L ratio = 1.3 (1.6 mm/1.2 mm)), subpentagonal, fore portion triangular, apex forming an obtuse angle (about 130°), both sides of the angle rectilinear, not reflexed upward, tip of triangle sharp; dorsal ocular area not small (dorsal ocular area was not visible through optical observation but micro-CT scanning revealed the presence of a patch which resembles a dorsal ocular area), dorsal interocular area about 10 times the maximum width of the dorsal ocular area; head dorsal surface with dense impressed coarse comma-shaped punctures, fore margin with a few very fine shallow transverse striae. **Pronotum:** subtrapezoidal, wider than long (W/L ratio = 1.5 (2.6 mm/1.7 mm)), wider than elytra, with base distinctly raised (a character normally associated with flightlessness); fore margin bisinuate, pronotal anterior carina (sensu Ballerio, 2021) feebly raised; fore angles obtuse; fore edge continuously finely margined, edges of sides with finely margined (dorsal view), base continuously strongly margined; base at middle protruding backwards; pronotal surface convex. Visible surface with dense strong punctation; punctures deep, mostly comma-shaped mixed to few horseshoe-shaped ones, their distance being less than their diameter. **Scutellum:** about as wide as long (W/L ratio = 1.1 (1.0 mm/0.9 mm)), sides proximally subparallel and distinctly notched by elytral articular process, then convergent to form a triangle with elongate acute apex and sides slightly curved inward. Surface slightly depressed in the middle, covered by dense impressed small transverse comma-shaped punctures. **Elytra:** longer than wide (W/L ratio = 0.9 (2.5 mm/2.7 mm)), apical fourth regularly rounded (dorsal view), apex slightly re-entering inward (lateral view); elytra regularly convex, although slightly flattened at disc; elytral suture raised all over its length, but more raised distally; humeral callus absent; elytral articular process distinct, smooth. Elytra densely punctured, basal and median third covered by a mix of large transverse irregular lines, large transverse impressed comma-shaped punctures and small transverse impressed comma-shaped punctures their distance being inferior than punctural diameter, pseudoepipleure covered by dense horseshoe-shaped punctures, their distance from each other being shorter than their diameter. **Legs:** protibiae straight, outer margin with one apical tooth visible; mesotibiae slender, thick; metatibiae triangular, elongate, flat; outer face of meso- and metatibiae with longitudinal and transverse deep lines.

#### Diagnosis

The presence of a feebly raised pronotal anterior carina (*sensu* Ballerio, 2021), pronotal and elytral disc punctation consisting of mainly comma-shaped punctures and the apparent presence of a dorsal ocular area are the combination of characters that allows to differentiate this species from all other known species of *Nesopalla*.

#### Remarks

Not having access to mouthparts and male genitalia, we attribute this new species to the genus *Nesopalla* Paulian & Howden, 1982^33^, mainly because of the distinctive raised base of the pronotum and the overall body shape. *Nesopalla succini* sp. nov. is clearly distinct from the other two known *Nesopalla*, i.e., *Nesopalla iviei* Paulian & Howden, 1982^33^, and *Nesopalla borinquensis* Paulian & Howden, 1982^33^. It differs from both in the presence of a pronotal anterior carina (*sensu* Ballerio, 2021) and the distinctive pattern of pronotal and elytral punctation. In *N. iviei*, both the pronotum and elytra have large ocellate punctures, while in *N. succini* sp. nov., the punctation consists of transverse comma-shaped punctures. In *N*. *borinquensis*, the dorsal punctation consists of fine anastomosing lines, and there are no comma-shaped punctures. Finally, in both *N. iviei* and *N. borinquensis*, the dorsal ocular area is absent. As indicated above, it is not possible to state without doubt that a dorsal ocular area is present in *N. succini* sp. nov. Actually, because of the imaging technique used (micro-CT scanning), we can see the presence of an eye, but this does not necessarily mean that such a structure is not concealed by the head cuticle (and therefore invisible under observation with an optical stereoscope). This is the first fossil *Nesopalla*, and the finding of such a beetle in amber is particularly remarkable since all known *Nesopalla* are flightless and the fossil species seems to also be flightless due to the strongly raised pronotal base and lack of elytral humeral callus (two characters usually linked to flightlessness in Ceratocanthinae).

#### Conglobation evolves with a single origin

From the strict consensus tree and related Bremer support at the main nodes (Supplementary Fig. 2), it is known that the extinct species (*Palaeopycnus circus* **sp. nov**., *Ceratocanthus huarongii* **sp. nov**. and *Nesopalla succini* **sp. nov**.) are far from the root of the tree and have good support (Bremer support 2) within the subfamily Ceratocanthinae. Specially, *Palaeopycnus circus* **sp. nov**. (Kachin amber) was close to the root of Ceratocanthini, *Ceratocanthus huarongii* **sp. nov**. belongs to the clade comprising the modern genus *Ceratocanthus* (Bremer support 2), and *Nesopalla succini* **sp. nov**. was close to the genera *Nesopalla*, *Afrocloetus* and *Cloeotus*.

The reported here fossils represent the earliest case of incomplete conglobation for scarab beetles and the earliest case of complete conglobation for insects (Fig. 1c, green and red stars). Herein, the new fossil records of *Palaepycnus* probably provide direct evidence that the incomplete conglobation in Ceratocanthinae probably originated at least in the Mesozoic and move the conglobation behaviour’s origination time in Scarab beetles back from the Cenozoic to the Early Cretaceous by ~70 Mya^15^.

Previously, phylogenetic analysis based on morphological characters led to the hypothesis that conglobation evolved in Ceratocanthinae only once^13^. While a recent study based on incomplete molecular data suggested that the conglobation of Ceratocanthinae originated from an ancestor with complete conglobation^15^. Here, mapping of the three conglobation archetypes onto phylogenetic trees reveals that the evolution of conglobation ability proceeded from a straight body to incomplete and then complete conglobation and supports the former hypothesis of single origin. In detail, conglobation (incomplete and complete) evolved without reversals in the common ancestor of Ceratocanthini (node 36), and complete conglobation evolved in the common ancestor of a subclade of Ceratocanthini consisting of *Ceratocanthopsis* + *Ceratocanthus* (node 64), with a single subsequent reversal in *Cloeotus* to incomplete conglobation (Supplementary Fig. 2).

#### Ceratocanthinae with higher defensive strength

**Fig. 3:**
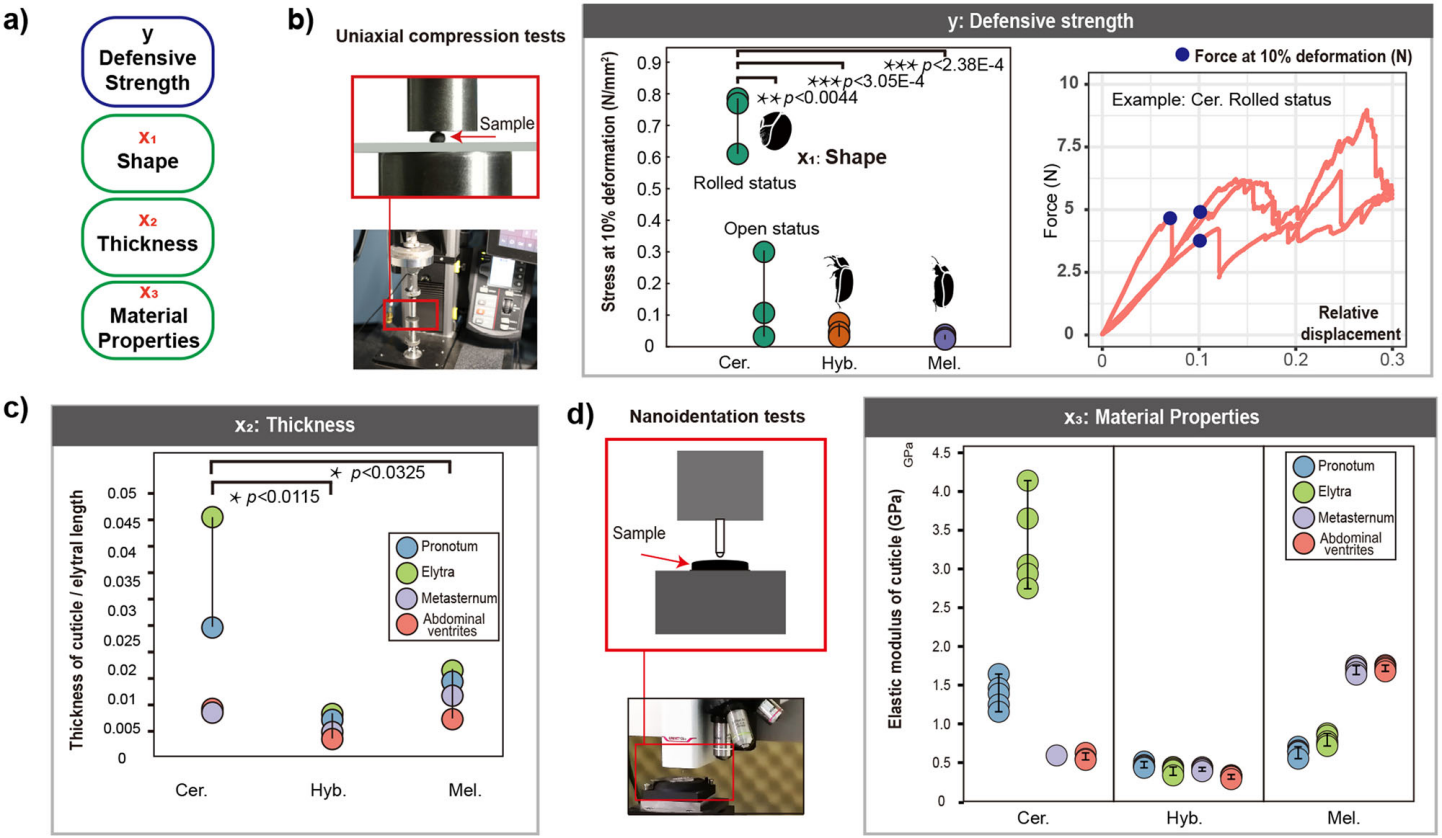
Functional morphological analysis of conglobation via quantitative measurements of mechanical properties of the body in representatives of Ceratocanthinae, Hybosorinae and Melolonthinae. **a** Central hypothesis and reasoning regarding the effects of shape, thickness and material properties on the defensive strength of beetles. Each *X* is an independent variable (cause), while y indicates the dependent variable (result). **b** Strength of the whole body determined by uniaxial compression tests (forces at 10% height deformation or at breaking points). **c** Ratio of thickness of cuticle to elytral length: four cuticles of the body wall (four coloured circles) measured by micro-CT scanning. **d** Material properties (elastic modulus) of four cuticles determined by nanoindentation tests. ∗*p* < 0.05, ∗∗*p* < 0.01, and ∗∗∗*p* < 0.001.

To quantify the defensive strength of scarab beetles, especially pill scarab beetles, and analyse how many factors contributed to this feature by employing an engineering approach, three species from Ceratocanthinae, Hybosorinae and Melolonthinae (belong to the same superfamily Scarabaeoidea) were studied (Fig. 3, Supplementary Note 2). The central hypothesis is that defensive strength was mainly determined by the shape, dimensions (material thickness), and material mechanical properties of the exoskeleton^34–37^ (Fig. 3a). For measuring the defensive strength, we simulated the common defensive scenario which beetles biting by greater sized attackers. Therefore, we estimated the defensive strength of exoskeletons by uniaxial compression tests of the whole body. Although there were variations between specimens in slope of curves for all species (Fig. 3b), Ceratocanthinae in rolled status obviously (*p* < 0.001 to other species, *p* < 0.01 to the open status) exhibited the highest resistance to deformation in terms of the force withstood by the samples (Fig. 3b). Hence, the defensive strength of rolled status Ceratocanthinae was considered to be the highest.

From the result of uniaxial compression tests, it is known that defensive strength of rolled status of Ceratocanthinae was obviously greater (*p* < 0.001) than the open status which proved that the spherical body are benefit to the higher defensive stress (Fig. 3b). For Ceratocanthinae, the advantages of conglobation are displayed also in some other scenarios: protect the soft parts of the body and made itself difficult to carry by attackers. After measuring the thickness and material properties of the cuticle, it was found that conglobate Ceratocanthinae with higher thickness and relatively stronger mechanical properties of the dorsal cuticle (Fig. 3c, d, Supplementary Table 2 and 3, Supplementary Fig. 6). In summary, Ceratocanthinae with higher defensive ability when biting by larger sized attackers, and the spherical shape, thickness of the cuticle and relatively higher material properties all contribute to this.

The respective living environments of Ceratocanthinae, Hybosorinae and Melolonthinae might provide some clues about why these beetles have different defensive strengths: the majority of Ceratocanthinae live in relatively hidden environments (such as termite nests), where they face direct biting from attackers such as soldier termites /ants and rapacious beetle larvae^13,38^. Their higher defensive strength could better protect them from hostile environments. Conversely, Hybosorinae (feeding on fresh carrion)^39^ and Melolonthinae (feeding on plants)^40,41^ live in relatively open habitats^38^. Therefore, they can escape direct predator attacks by flying away or dropping to the ground^42,43^.

To the best of our knowledge, we made the first attempt to quantify the mechanical defensive strength in scarab beetles^44^. While there might be systematic biases and/or simplifications in the quantification approaches—micro-CT, nanoindentation, and uniaxial compression for the thickness, material property, and defensive strength evaluations, respectively—they were negligible for drawing our qualitative conclusions^45–47^ (Supplementary Note 2). Therefore, our integrated study proved that in Ceratocanthinae the allometric dorsal body wall thickness, the material mechanical properties of the cuticle and spherical shape all contribute to the higher defensive strength of this subfamily when bitten by attackers (Fig. 3). We anticipate that future studies of candidate regulatory gene expression in the cuticle of Ceratocanthinae will contribute to our understanding of the variation in material properties and thickness^48^.

#### Morphological characters adapted for conglobation

Unlike other animals displaying conglobation behaviours, Ceratocanthinae are the morphologically unique arthropods able to form a tight ball through a process that involves three body trunk segments and tibiae. Hence, we firstly examined the geometry of the three main body trunk segments involved in the conglobation process (head, prothorax and mesothorax). More precisely, the analysis was conducted by using the shape of the head in dorsal view, pronotum in lateral view and elytra in lateral view as the main indicators of adapted conglobation capability.

**Fig. 4:**
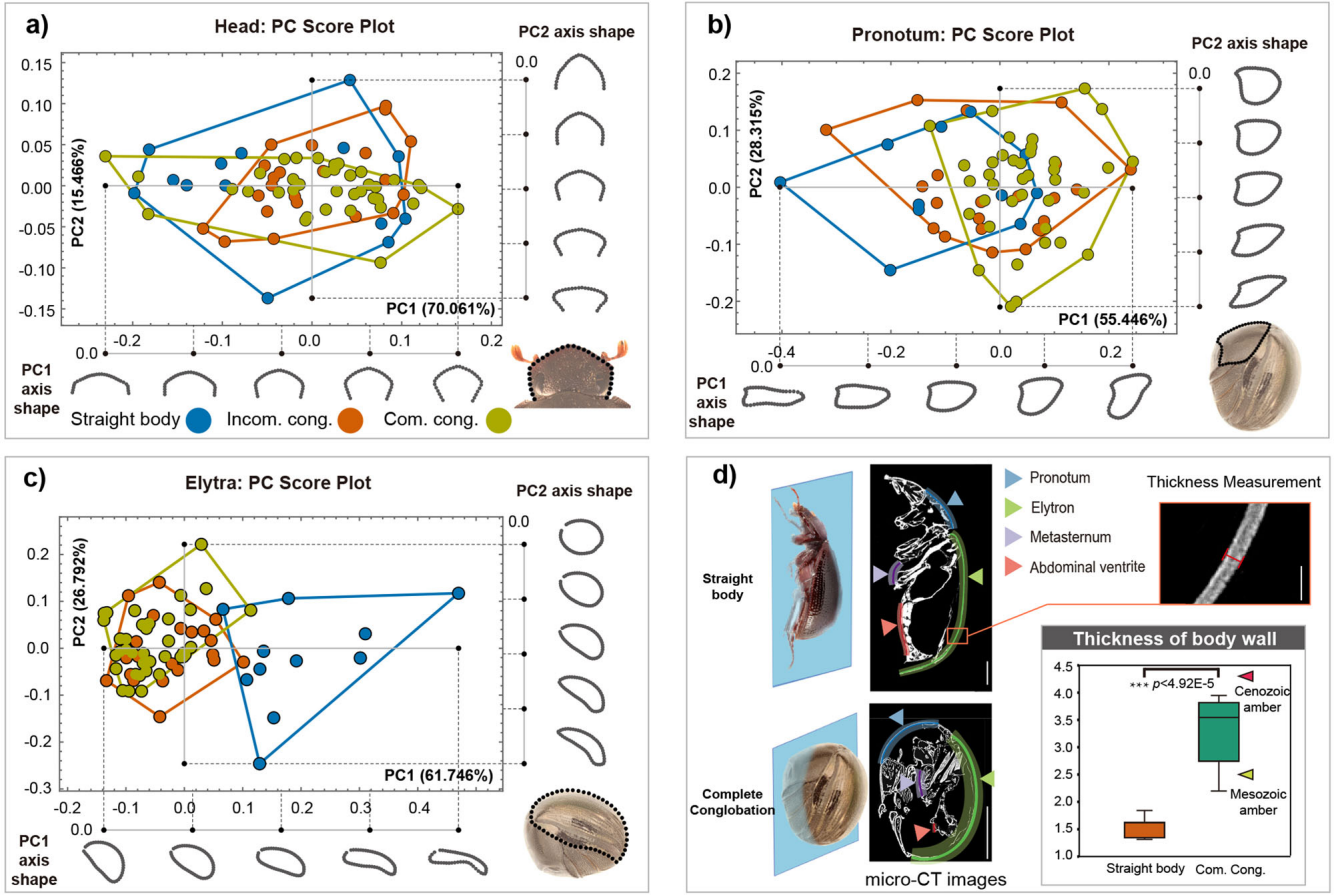
Morphological analysis of conglobation-related characters in Ceratocanthinae. **a**–**c** Geometric morphometric analysis of basic shapes: **a** head; **b** pronotum; and **c** elytra. The PC score plot shows the distribution of species with three archetypes based on the morphological variation in test traits along principal component axes (PC1 and PC2). The PC1 and PC2 axis shapes show deformations at certain positions along the axes. The figure in the lower right-hand corner shows landmarks describing the basic shape of the head, pronotum and elytra used in the geometric morphometric analysis. **d** Morphometric analysis of micro-CT images of body wall thickness in species of scarab beetles (Scarabaeoidea) with straight bodies or complete conglobation (Supplementary Table 2). The box plot shows the ratio of outer (elytra plus the pronotum) to inner (abdominal ventrites plus the metasternum) surface thickness. Scale bars in micro-CT images: 1 mm; Scale bars in thickness measurement image: 0.1 mm. Green–yellow triangle: *Palaeopycnus circus* sp. nov. from Mesozoic amber, red triangle: *Nesopalla succini* sp. nov. from Cenozoic amber. ∗∗∗: *p* < 0.001.

We observed that (a) there was noticeable variation between incomplete conglobation and complete conglobation (principal component analysis, PCA), especially in relation to the shape of the head and pronotum, and (b) the main variation in the elytra occurred between the straight-body and the conglobation archetypes (PCA). More precisely, the shape of the anterior margin of the head varied from subrectangular to triangular (Fig. 4a, PC2), and the shape of the pronotum in lateral view varied from long and less convex in species with incomplete conglobation archetypes to short and more convex in species with complete conglobation (Fig. 4b, PC1). The shape of the elytra in lateral view varied from less convex in species with straight-body archetypes to more convex and greatly curved in species with complete conglobation (Fig. 4c, PC1, PC2). Based on the character differences revealed by PCA, canonical variate analysis (Supplementary Fig. 7, CVA) confirmed that the geometries of the head, pronotum and elytra were significantly different between the three archetypes (*P*-values of Mahalanobis distances among the three archetypes all <0.0001, based on 10000 permutation rounds).

**Fig. 5:**
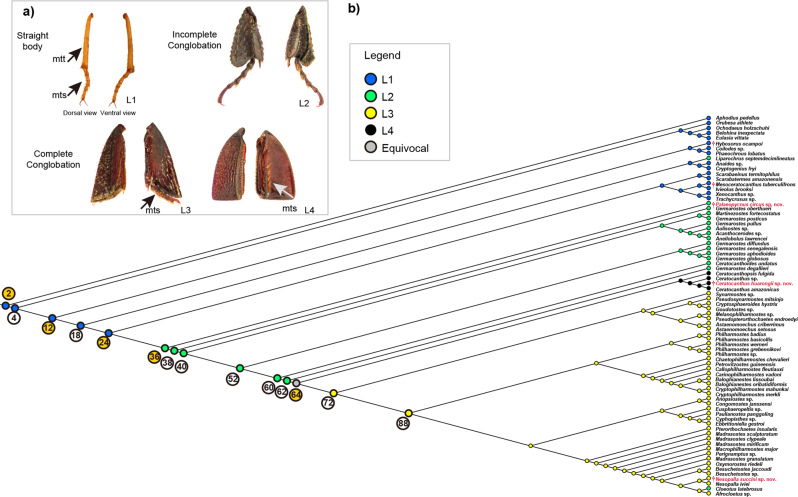
Evolution of metatibiae in Ceratocanthinae. **a** Comparative morphology of the metatibiae; mtt: metatibia, mts: metatarsus. L1: rod-like metatibia (*Ivieolus brooksi*), L2: unconstrained triangular metatibia (*Germarostes globosus*), L3: interlocking triangular metatibia (*Astaenomoechus setosus*), L4: interlocking triangular metatibia with a tarsal groove (*Ceratocanthus amazonicus*). **b** Ancestral state reconstruction of the metatibiae.

Then we examined the geometry of the metatibia. The metatibiae of straight-bodied Ceratocanthinae were elongate and narrow with an approximately rectangular section (Fig. 5a, L1), while the legs of Ceratocanthinae with conglobation were shorter and triangular (Fig. 5a, L2–L4). The metatibiae of species with incomplete conglobation archetypes did not show any interlocking mechanism and did not match the mesotibiae once the beetle had conglobated, whereas the metatibiae of species with complete conglobation archetypes possessed interlocking devices that could match them with the mesotibiae to form a perfect, tight ball. Additionally, for two genera of Ceratocanthinae with complete conglobation (*Ceratocanthus* and *Ceratocanthopsis*), the metatibiae showed a groove along the inner side where the tarsi were concealed once the beetle conglobates (Fig. 5a, L4). The analysis of leg shapes suggested that the largest change occurs in the transition from incomplete to complete conglobation which is supported by ancestral reconstruction (Fig. 5b, node 64). The Mesozoic fossils showed leg morphology somewhat similar to that of extant Ceratocanthinae with incomplete conglobation, such as the genus *Acanthocerodes* and some species of *Germarostes*. If we assume that *Palaeopycnus* gen. nov. represents a basal lineage of Ceratocanthinae, then the recent hypothesis of Grebennikov & Smith^15^ (supported by molecular data) that ancestral Ceratocanthinae had complete conglobation is unsupported, and the hypothesis resulting from the tree generated by Ballerio & Grebennikov^13^, which stipulated that the ancestral Ceratocanthinae had incomplete conglobation, is supported.

Additionally, a micro-CT analysis was performed to examine the thickness of the Scarabaeoidea exoskeleton in order to elucidate the mechanics and advantages of conglobation behaviours (See Result and discussion part of *Ceratocanthinae with higher defensive strength*). Here, we found that the exoskeleton of species in Certocanthinae with conglobation had a thickened pronotum and elytron but thinner abdominal body parts (Fig. 4d), whereas the pronotum, elytra and abdominal parts of species without conglobation showed more similar thickness (Fig. 4d, *p* < 0.001). The micro-CT measurements in representative species (Supplementary Table 2 and Fig. 4d) demonstrated that the species with conglobation had a greater ratio of outer surface to inner surface thickness than the straight-bodied species, which was most evident in the elytra and abdominal ventrites (Supplementary Fig. 8). This allometric phenomenon of redistributed body wall materials might be driven by a trade-off between different energy allocation strategies^49,50^. Specifically, when the total amount of body wall material could not be increased obviously, more body wall material was distributed to the dorsal body wall, i.e. outer surface of the ball. Notably, the body wall of both the Mesozoic (*Palaeopycnus circus* sp. nov.) and Cenozoic (*Nesopalla succini* sp. nov.) Ceratocanthinae displayed allometry in exoskeleton thickness (Fig. 4d green–yellow and red triangles), and the differences were more obvious in *Nesopalla succini* sp. nov. This suggests that the redistribution of body wall material is gradual, corresponding to the evolution of conglobation behaviour in history.

#### Adaptive characters probably experienced different evolutionary histories

**Fig. 6:**
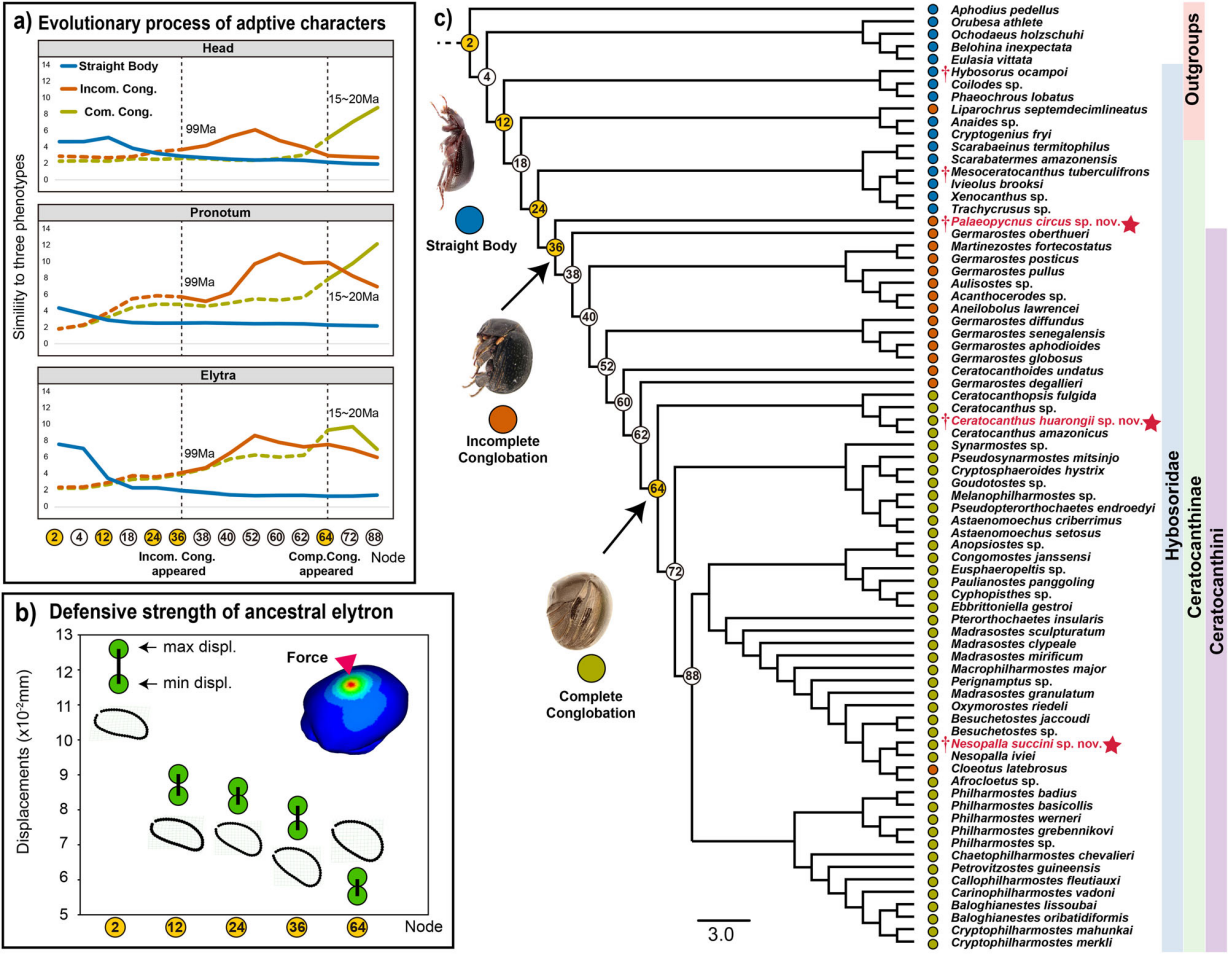
Ancestral state reconstruction of key nodes illustrates the evolutionary process of characters associated with conglobation adaptation. **a** Trend line of similarities of the reconstructed shapes at key nodes to the three archetypes (straight body, incomplete conglobation and complete conglobation). The similarities are represented by reciprocals of normalized distances (the sum of distances to the three archetypes normalized as 1). **b** The result of finite element analysis (FEA) of the reconstructed elytron at selected representative nodes (nodes 2, 12, 24, 36 and 64); higher displacement means lower defensive strength. **c** The topology of the phylogenetic tree derived from the first of the five most parsimonious trees obtained in the phylogenetic analysis. Terminals are coloured according to the three conglobation archetypes (blue circles represent a straight body, orange circles represent incomplete conglobation, and yellow–brown circles represent complete conglobation). Red stars for the terminal taxa indicate the extinct taxa newly described in this article. Numbers within circles represent the ancestral nodes; orange circles indicate the selected representative nodes (see Methods).

To investigate the evolutionary process of these geometric characters (shape of the head, pronotum and elytra) contributing to conglobation, we measured the similarity of the values of each character at a series of ancestral nodes (nodes 2, 4, 12, 18, 24, 36, 38, 40, 52, 60, 62, 64, 72, and 88) to those of the three extant archetypes (straight body, incomplete conglobation, complete conglobation), based on the corresponding PCA embeddings (see Methods) (Fig. 6c). We found that the changes in the shape of the head, pronotum and elytra exhibited overall similar trends: the similarity of ancestral nodes to the straight-body archetype decreased through time (Fig. 6a, blue line), while the similarity to incomplete and complete conglobation archetypes increased (Fig. 6a, orange and yellow–brown lines). However, the three characters have evolved independently in the temporal process of adaptation for conglobation. Before the emergence of the incomplete conglobation archetype at node 36 (99 Ma), the elytra shape likely underwent a relatively rapid decrease in similarity to the straight body archetype, potentially contributing to the formation of the dorsal half of the ball as a key step in obtaining partial conglobation ability. The pronotum followed the elytra but together with a relatively rapid increase in similarity to incomplete conglobation archetype at node 52, while head shape did not show much of an increase in similarity to conglobation archetypes until complete conglobation appeared (at approximately node 64, 15–25 Ma). It seems that this was the last step in the evolutionary process leading to a nearly complete spherical structure.

From the result of ancestral reconstruction, it is clear that when nodes are closer to the tips of the tree, the elytral shapes are more similar to the complete conglobation archetype. To learn more about the evolution of defensive strength, we analysed the simulated 3D model of the elytron at several key nodes (See Methods) through finite element analysis (FEA), which showed a trend of greater defensive strength in terms of the gradually lower both maximum and minimum displacements when nodes were close to the tips of the tree (Fig. 6b). These results indicated that the spherical shape of elytra improved the defensive strength of pill scarab beetles (Ceratocanthinae).

#### “Attackers stress” may drive the evolution of conglobation

The capability of Ceratocanthini to roll the body into a ball might have evolved to support a lifestyle in the hostile environment of termite nests/leaf litter/rotten wood, all those environments are relatively hidden and narrow and under the constant pressure of attackers such as soldier termites and occasionally invading ants, other predatory insects, and other arthropods. Provided the defensive benefits of conglobation, we propose the “attackers stress” hypothesis to explain why conglobation behaviours originated in pill scarab beetles. The conglobation behaviour has always been seen as a defensive behaviour to make the body more compact and less easy to be attacked by detaching legs, this is the case of Isopods^51^, and some beetles such as Histeridae and Byrrhidae.

Incomplete conglobation occurs in basal genera of Ceratocanthini, all of them living in leaf litter. It could be therefore hypothesized that the event triggering the appearance of the conglobating morphological features of Ceratocanthini was the need to defend themselves from attackers living in leaf litter or under bark (ants, ground beetles, etc.). Complete conglobation conversely occurs in more advanced Ceratocanthini, which sometimes are found inside termite nests. It could be therefore hypothesized that the conglobation capability of Ceratocanthinae was a preadaptation that made them able to penetrate termite nests where this feature protected them both from the attacks of termites and from the attacks of invading ants. Among the few known termite hosts of Ceratocanthini there are the Mastotermitidae (originated during the Mesozoic) and the Rhinotermitidae and Termitidae (both originated during Cenozoic)^52,53^. It could be also hypothesized that during the Mesozoic, the social caste differentiation of termites was still primitive, with potentially less powerful soldiers, and perhaps incomplete conglobation was sufficient to face the hostile stress. Along with the success of clearly defined social castes of termites in Rhinotermitidae and Termitidae, which may present more threatening soldier attacks^54^, the pill scarab beetle species capable of complete conglobation would have gained more advantages. A similar situation also existed in ants^55^. The morphological plasticity found within Ceratocanthinae allowed also to develop another completely different strategy for living in termite nests, which is found in Scarabatermitini and Ivieolini, involving morphological adaptations including physogastry and depigmentation, mimicking morphology observed in many termite-associated insects^13,16^.

It is also possible to hypothesize that conglobation can be involved in a behaviour evolved for “rolling away”: some species of Ceratocanthinae having adapted to life in the canopy, after conglobation could roll away when disturbed. However, since all basal Ceratocanthinae live in leaf litter or rotten wood, then it is unlikely that the “rolling away” hypothesis could have played a role in shaping the morphology of Ceratocanthinae, this hypothesis could simply stand as a kind of secondary adaptation. The factors driving conglobation behaviours are probably more complex and need further comprehensively research.

## Methods

### Specimens

The specimens of Kachin amber were derived from amber deposits in the Hukawng Valley of Myanmar. We tentatively followed the age (98.8 ± 0.6 million years, Cenomanian, earliest Late Cretaceous) given by U–Pb dating of zircons from the volcaniclastic matrix of the amber^56,57^. The specimens of Dominican amber were derived from amber deposits in the Dominican Republic, which date to 15 to 20 million years ago^58^. Type specimens are currently housed in the Institute of Zoology, Chinese Academy of Sciences, Beijing, China (IZAS) (Ming Bai: baim@ioz.ac.cn) (details see Materials of every species)(Supplementary data 2). The specimens of extant species of Scarabaeidae are deposited in the IZAS and personal collections of Alberto Ballerio (alberto.ballerio.bs@aballerio.it).

Observations were carried out under an Olympus SZ61 stereomicroscope. The digital images were taken with a Canon 5D digital camera in conjunction with a Canon MP-E 65 mm f/2.8 1-5X Specimens were also examined and photographed under Nikon SMZ25 microscope, with an attached Nikon DS-Ri2 digital camera system. Macro Lens fitted to a macro rail (Cognisys). Photomicrographs of *Palaeopycnus circus*, *Palaeopycnus fushengii* were taken using a Zeiss confocal laser scanning microscope (Zeiss, LSM780) equipped with 10× objective, and those with red and green backgrounds used fluorescence as a light source.

### Nomenclatural acts

This published work and the nomenclatural acts it contains have been registered in ZooBank, the online registration system for the International Code of Zoological Nomenclature (ICZN). The ZooBank LSIDs (Life Science Identifiers) can be resolved and the associated information viewed through any standard web browser by appending the LSID to the prefix “http://zoobank.org/”. The LSIDs for this publication are: urn:lsid:zoobank.org:act:FC9EFB6C-5345-441B-860B-CBB369B59685; urn:lsid:zoobank.org:act:277AC424-C435-4524-A2D4-864920BDF2F5; urn:lsid:zoobank.org:act:E439A7D8-5125-459F-A6C6-7456F2989552; urn:lsid:zoobank.org:act:00AC0E49-1ED8-4C3E-BB10-DF6FCE964D9C; urn:lsid:zoobank.org:act:82C7EFA6-F512-4686-8FDE-319136F3EBB7.

### Micro-CT scanning and 3D reconstruction

The specimens were scanned with a Micro-XCT 400 (Carl Zeiss X-ray Microscopy, Inc., Pleasanton, USA) at the IZAS. Based on the obtained image stacks, the structures of the specimens were reconstructed and segmented with Amira 5.4 (Visage Imaging, San Diego, USA). Subsequent volume rendering and animation were performed with Geomagic Studio 2013 (Geomagic Inc., USA). Due to preservation, not all parts of the beetles were reconstructed.

### Phylogenetic analysis of Ceratocanthinae

Morphological characters and their states are shown in Supplementary Note 1 and Supplementary Data 1. Seventy-six taxa of Scarabaeoidea were selected, including (i) five species of non-Hybosoridae families and six species from Hybosoridae but not Ceratocanthinae as outgroups and (ii) three new species (*Palaeopycnus circus*, *Ceratocanthus huarongii* and *Nesopalla succini*), one fossil of Ceratocanthinae (*Mesoceratocanthus tuberculifrons*), and the species from Ballerio & Grebennikov^13^ (61 extant species of Ceratocanthinae, 56 of which were Ceratocanthini). Due to the limitations of fossils, some characters were not clearly visible in *Palaeopycnus fushengii*, which was not included in phylogenetic analysis in this study, but there is enough evidence proving that *Palaeopycnus circus* and *Palaeopycnus fushengii* belong to the same genus (see Remarks on these species). Thus, in the genus *Palaeopycnus*, we selected only *Palaeopycnus circus* for phylogenetic analysis.

Maximum parsimony (MP) analysis with equal weighting (EW) was executed with TNT version 1.5 software (Goloboff & Catalano, 2016), using the settings Analyze > ‘traditional search’; ‘max. Tree’ = 10,000; ‘random seed’ = 100; ‘number of additional sequences’ = 1,000; and ‘trees to save per replication’ = 1. Furthermore, ‘tree bisection reconnection (TBR)’ was used as the permutation algorithm for the branches. All characters were treated as unordered and nonadditive. The MP analysis yielded the five most parsimonious trees. A strict consensus tree was saved based on MPT trees in WinClada (tree length = 564 steps, CI = 0.21, RI = 0.67). Nodal support was assessed by determining Bremer support for up to ten suboptimal trees. Morphological characters were optimized with parsimony on the strict consensus tree (Supplementary Fig. 2), showing only unambiguous changes. Black circles indicate nonhomoplasious changes, and white circles indicate changes in homoplasious characters. Numbers above branches represent character numbers.

### Deformation resistance and defensive strength evaluation of the exoskeleton

Uniaxial compression experiments were conducted by Instron 5943 to quantify the deformation resistance and defensive strength of the three groups of beetles (*n* = 3) (the same species as in the nanoindentation tests). The height of the samples was measured with a micrometre precision before the samples were compressed at a constant displacement speed (250 μm/min) in a prone position until ~50% deformation of the original height. Force-normalized deformation (the displacement divided by the original height) curves were used to offset the size variation between species. The force at 10% deformation (or at the breaking point where the force instantly dropped by >5% from the maximum force) was chosen to represent the defensive strength of the exoskeleton and compared between groups. To exclude the effect of size, the force was divided by the projected area.

### Material property analysis of the cuticle

Prior to mechanical testing, adult dry scarab beetles deposited in collections (IZAS) were dissected. Pieces of structures were separated from the specimens. They were fixed on the specimen holder, using adhesive tape. To measure the elastic moduli of the beetles’ exoskeletons, nanoindentation tests were performed by using an UNHT3 Bio instrument (Anton Paar, Austria) with a Berkovich indenter. The testing parameters (Acquisition rate = 50.0 Hz, Max load = 10.00 mN, Loading rate = 100.00 mN/min) were optimized to match the mechanical properties of the samples (indentation depth~ 2 μm). The elastic moduli were calculated by using the Pharr and Oliver model^59^, and the Poisson ratio of the cuticle was taken as 0.3^60^. Four parts of the body wall (pronotum, elytra, metasternum and abdominal ventrites) of the three groups of beetles (one specimen per group), namely, Ceratocanthinae with complete conglobation (*Pterorthochaetes* sp.), straight-bodied Hybosoridae (*Phaeochrous* sp.) and straight-bodied Melolonthinae (*Apogonia* sp.), were measured with five repetitions (the spacing distance of repetitions about 50 μm) at radial direction.

### Morphometric analysis

The head, pronotum and elytra were analysed by a geometric morphometric method, while the body wall were analysed by a general morphometric method, as described in detail below.

Geometric morphometric analyses of head, pronotum and elytra variation were based on single curve marks from 74 species’ dorsal and lateral view images (Fig. 4a–c). The curves were resampled at 30, 50 and 50 semilandmarks of the head, pronotum and elytra, respectively (Fig. 4a–c). Cartesian coordinates of the landmarks and curves were digitized by using tps-DIG 2.05^61^, and the landmark configurations were scaled, translated, and rotated against the consensus configuration using the Procrustes superimposition method^61^. The geometric differences in the head, pronotum and elytra among the three different archetypes (straight body: 14 species, incomplete conglobation: 22 species and complete conglobation: 38 species) were analysed by PCA and canonical variate analysis (CVA) in MorphoJ 1.06a^62^.

The thickness of the exoskeleton was measured based on the micro-CT scan data of representative species in Scarabaeoidea (Supplementary Table 2). The pronotum and abdominal ventrites were measured along the central longitudinal plane of the body, and the metasternum and elytra were measured along the quarter longitudinal plane of the body. The thickness measurements were averaged (*n* = 3) along the longitudinal plane (Fig. 4d).

### Ancestral character reconstruction

Because some nodes within extant genera were collapsed in the strict consensus tree, they could not be used to trace the ancestral history of characters. Therefore, tree 1 from TNT, which had basic topology similar to that of the strict consensus tree, was chosen to reconstruct the status of nodes. The shapes of the head, pronotum, and elytra for each species from the Ceratocanthinae and the other high-ranking taxon from the Scarabaeoidea were mapped onto the calculated phylogenetic tree. Landmark data were entered into Mesquite 3.61 (build 927) (1997–2019 W. Maddison and D. Maddison) as a continuous matrix and linked to the phylogenetic tree. As branch lengths were missing in this tree, we used similar species instead (Supplementary Table 4). All branches were assigned an equal length (i.e., assuming an evolutionary model with the same expected degree of morphological change on all branches). The ancestral forms at all nodes were reconstructed using the traces of all characters and the landmark drawings from the modules of the Rhetenor package in Mesquite. Then, the ancestral states at all nodes were inferred and exported.

The computed data for the ancestral nodes were integrated with the original landmark data. Every ancestral node was considered an independent group, and terminal nodes were divided into corresponding archetypes (straight body, incomplete conglobation and complete conglobation). In this case, the shape differences among ancestral nodes and terminal nodes were inferred based on PCA and CVA in MorphoJ 1.06a^62^. The conglobation archetypes of ancestors (nodes) were evaluated based on normalized Mahalanobis distances (the sum of Mahalanobis distances to the three archetypes normalized as 1), and the similarities to the three archetypes were represented by reciprocals of normalized distances.

The metatibia states were divided into four options (L1–L4) and linked to the same tree mentioned above, and then the ancestral states at all nodes were reconstructed in Mesquite 3.61. The ancestral shapes (nodes) were computed and compared with the shapes of modern species with the three conglobation archetypes.

### FEA simulation of the ancestral elytra

An FEA approach was applied to evaluate the defensive strength of the elytra at different nodes. From the result of ancestral character reconstruction, the lateral view of the elytron at different nodes was inferred. The selected representative nodes were node 2 (earliest ancestor in this tree), 12 (ancestor of family Hybosoridae), 24 (ancestor of subfamily Ceratocanthinae), 36 (ancestor of tribe Ceratocanthini) and 64 (ancestor of species with the complete conglobation archetype). However, the 3D morphology of these “specimens” was not preserved. It was not possible to infer the functional morphology directly. Therefore, we first created a 3D model based on extant pill scarab beetles. Then, the models were modified to fit the lateral view of the elytron at different nodes using 3Dmax 2021 (Autodesk). The surface files were imported into Z88Aurora V3 (http://www.z88.de/) for FEA. A tetrahedral volume mesh was produced using the NETGEN setting, with tetrahedrons (linear) and a value of 5. The force loads were applied to the tip of the elytron, and constraints were set throughout the bottom of the elytron. The elastic, linear and homogeneous material was set. The SORCG setting with von Mises stress failure theory was selected in the solver step. The figures of the stresses at corner nodes were saved in the postprocessor step. The material properties were consistent with the results of nanoindentation tests, with an average elytral elastic modulus of 3310.26 MPa. A Poisson ratio of 0.3 was measured for the lobster cuticle, and we used this value in our simulations^60^. We chose a density value of 0.5 g cm^−3^ in this study^63^.

### Statistics and reproducibility

The sample size and number of replicates for each experiment are noted in the respective section describing the experimental details. The *p-*values were calculated by two-tails *t*-test.

### Reporting summary

Further information on research design is available in the Nature Research Reporting Summary linked to this article.

## Supplementary information

Supplementary Information

Description of Additional Supplementary Files

Supplementary Data 1

Supplementary Data 2

Reporting Summary

## Data Availability

The datasets generated during and/or analysed during the current study are available from the corresponding author on reasonable request. The extinct and extant specimens are available from the deposited museums.
