# Supplementary material for: The evolution of conglobation in Ceratocanthinae
Source: Commun Biol. 2022 Aug 6;5:777. doi: 10.1038/s42003-022-03685-2 (PMC9357020; doi:10.1038/s42003-022-03685-2)
Supplement: Supplementary file 1 — Supplementary Information [file 42003_2022_3685_MOESM1_ESM.pdf]

Supplementary Materials for

The evolution of conglobation in Ceratocanthinae

Yuanyuan Lu[1], Alberto Ballerio[2], Shuo Wang[3,4], Zhengting Zou[1,5], Stanislav N. Gorb[6], Tao Wang[7,8], Lulu Li[1], Shen Ji[3,4], Zhengyu Zhao[1,5], Sheng Li[1], Yijie Tong[1], Yandong Chen[1], De Zhuo[9], Cihang Luo[5,10], Weiwei Zhang[11], Ning Liu[1], Qi Gu[3,4,5]*, Ming Bai[1,5,12]*

* Corresponding author: M. Bai (email: baim@ioz.ac.cn); Q. Gu (email: qgu@ioz.ac.cn)

This PDF file includes:

Supplementary Note 1 to 2
Supplementary Figures 1 to 8
Supplementary Tables 1 to 4
Supplementary References

Supplementary Note 1. Morphological characters and their states

(partly following Ballerio & Grebennikov 2016)

0. Body, horseshoe-shaped punctures on dorsum: absent = 0; present = 1.
1. Body, capacity of conglobation by deflexing head and pronotum: absent = 0; present, even if incomplete = 1.
2. Body, enrollment coaptations (ability to make a perfect ball): absent = 0; present = 1.
3. Body, exoskeleton: fully pigmented = 0; depigmented = 1.
4. Head capsule, pentagonal shape in dorsal view: absent = 0; present = 1.
5. Shape of clypeus: semicircle = 0; triangle = 1; rectangle = 2.
6. Head capsule, length/width ratio in dorsal view: less than one = 0; more than one = 1.
7. Head capsule, clypeus, anterior serration: absent = 0; present = 1.
8. Head capsule, labrum and clypeus, whether on same plane in lateral view: yes = 0; no = 1.
9. Head capsule, vertical dimension of apical clypeal extremity: absent = 0; present = 1.
10. Head capsule, vertical dimension of apical clypeal extremity, as compared to one fourth clypeal length: smaller = 0; greater = 1.
11. Head capsule, externally visible genal canthus: absent = 0; present = 1.
12. Head capsule, genal canthus, whether reaching postocular area: no = 0; yes = 1.
13. Head capsule, fore margin with a distinct angle delimiting genae: absent = 0; present = 1.
14. Head capsule, genal suture: absent = 0; present = 1.
15. Head capsule, genal suture generating a slight discontinuity in the fore margin: absent = 0; present = 1.
16. Antennae, number of antennomeres: 10 = 0; 9 = 1; 8 = 2; 7 = 3.
17. Antennae, antennomere 3: wider than long = 0; as long as wide = 1; longer than wide = 2.
18. Antennae, each of antennomeres 4 to 7, proportion: wider than long = 0; as long as wide = 1; longer than wide = 2.
19. Antennae, club length as compared to that of funicle: shorter = 0; subequal = 1; longer = 2.
20. Antennae, proximal club antennomere encapsulating rest of the club: absent = 0; present = 1.
21. Antennae, proximal club antennomere, setae on proximal face: absent = 0; present = 1.
22. Antennae, proximal club antennomere, setae on proximal face covering: whole surface = 0; only proximal area = 1; only distal area = 2.
23. Mandibles, conjunctive (sensu Nel & Scholtz 1990): absent = 0; present = 1.
24. Mandibles, ventral pore on basal part: absent = 0; present = 1.
25. Mandibles, apical part, number of teeth in addition to mandibular apex: 0 = 0; 1 = 1; 2 = 2.
26. Mandibles, mesal brush very developed with mandibular apex not exceeding it: absent = 0; present = 1.
27. Maxillae, length of distal palpomere compared to that of two preceding: shorter = 0; subequal = 1; longer = 2.
28. Maxillae, sclerotization of galea: absent = 0; present = 1.
29. Labium, medial notch on anterior edge: absent = 0; present = 1.
30. Labium, length of distal palpomere compared to that of two preceding: longer = 0; shorter = 1.
31. Labium, lateral expansion of third palpomere making it dissimilar to others: absent = 0; present = 1.
32. Labium, palpomeres, number: 3 = 0; 4 = 1.

33. Labrum, distal longitudinal furrow, even if shallow: absent = 0; present = 1.
34. Labrum, anterior truncation: absent = 0; present = 1.
35. Labrum, surface: smooth = 0; wrinkled = 1; granulose = 2; punctate = 3.
36. Labrum, apical fringe: absent = 0; present = 1.
37. Epipharynx, longitudinal carina on median process: absent = 0; present = 1.
38. Epipharynx, sclerotization: absent = 0; present = 1.
39. Epipharynx, setae on median process: absent = 0; present = 1.
40. Prothorax, prosternal apophyses, whether reaching inner wall of pronotum [internal view, dissection re-quired]: not reaching = 0; reaching = 1.
41. Prothorax, basisternum, longitudinal crest: absent = 0; present = 1.
42. Prothorax, basisternum, deep transverse anteriorly opened depression: absent = 0; present = 1.
43. Prothorax, sternellum, ventral projection: absent = 0; present = 1.
44. Prothorax, lateroventral expansion of hypomeron: absent = 0; present = 1.
45. Pronotum, swollen anterior margin: absent = 0; present = 1.
46. Pronotum, swollen posterior margin: absent = 0; present = 1.
47. Pronotum, anterior pronotal angles: acutely pointed = 0; broadly rounded = 1; truncate = 2.
48. Pronotum, posterior median swelling: absent = 0; present = 1.
49. Pronotum, embossed sculpturing: absent = 0; present = 1.
50. Pronotum, vestigial hind angles: absent = 0; present = 1.
51. Thoracic dorsum, distal part of exposed scutellum, sides: markedly convex forming rounded obtuse apex = 0; weakly convex forming lanceolate apex = 1; weakly concave forming acutely pointed apex = 2.
52. Thoracic tergite, mesotergite, metascutal furrow, posterior projection beyond posterior metatergite edge: absent = 0; present = 1.
53. Thoracic tergite, mesotergite, length compared to that of elytra: 1/4 the length of elytra = 0; 1/3 the length of elytra = 1.
54. Thoracic venter, metaventrite: rectangular = 0; triangular = 1.
55. Elytra, longitudinal striation on dorsal side: absent = 0; present = 1.
56. Elytra, area between striated articular area and inferior sutural stria (“marginal area” sensu Paulian 1977): absent = 0; present = 1.
57. Elytra, extension of sutural stria continuing from elytral apex along elytral lateral sides (“inferior sutural stria” sensu Paulian 1977): absent = 0; present = 1.
58. Elytra, striated articular area (sensu Paulian 1977): absent = 0; present = 1.
59. Elytra, sutural stria: absent = 0; present (incomplete) = 1; present (complete) = 2.
60. Metathoracic wings: absent = 0; present = 1.
61. Metathoracic wings: vestigial, < 30% elytral length = 0; short, about 100% elytral length = 1; long, about 200% elytral length = 2.
62. Wings, vein MP4, length relative to half length of CuA: shorter = 0; longer = 1.
63. Wings, distal part of vein MP4: straight and parallel to CuA = 0; bent towards CuA = 1; joining apically CuA = 2.
64. Wings, vein CuA, distal fork: absent = 0; present = 1.
65. Wings, vein MP3: absent = 0; present = 1.
66. Wings, loop of vein RP MP 1+2: absent = 0; present = 1.
67. Wings, sinuation of vein AA: even = 0; at right angle = 1.

68. Wings, distal expansion of vein MP1+2: absent = 0; present = 1.
69. Wings, short proximal expansion of vein CuA3+4: absent = 0; present = 1.
70. Wings, vein RA3, vertical secondary sclerification at base: absent = 0; present = 1.
71. Legs, procoxae, orientation of longest axle: horizontal = 0; vertical = 1.
72. Legs, protibiae: straight = 0; curved = 1.
73. Legs, protibiae, dentation on distal third of outer side: absent = 0; present = 1.
74. Legs, protibiae, sexual dimorphism in shape: absent = 0; present = 1.
75. Legs, female protibiae, apically elongate: absent = 0; present = 1.
76. Legs, female protibiae, pointed: absent = 0; present = 1.
77. Legs, female protibiae, with longer apical tooth: present = 0; absent = 1.
78. Legs, protibiae, longitudinal carina on ventral side: absent = 0; present = 1.
79. Legs, protarsi, widening of male tarsomeres: absent = 0; present = 1.
80. Legs, meso- and metafemora, distal emargination on posterior edge: absent = 0; present = 1.
81. Legs, meso- and metatarsi, capability to be folded along the inner side of tibia: absent = 0; present = 1.
82. Legs, meso- and metatibiae in cross section: rounded = 0; parallel sided = 1.
83. Legs, mesotibiae, transverse carinae on outer surface: absent = 0; present = 1.
84. Legs, mesotibiae, number of apical spurs: one = 0; two = 1.
85. Legs, mesotibiae, inner apical spur in males: straight = 0; curved = 1.
86. Legs, posterior angle of metatrochanter, posterior projection beyond posterior edge of metafemora: absent = 0; present = 1.
87. Legs, metatibiae: subrectangular = 0; triangular = 1.
88. Legs, metatibiae, apical corbel (sensu Thompson 1992): absent = 0; present = 1.
89. Legs, protarsi, length of female first tarsomere, to that of all others: distinctly shorter = 0; subequal = 1; distinctly longer = 2.
90. Legs, protarsi, tarsal insertion at: middle = 0; distal third = 1; apex = 2.
91. Legs, protibiae, proximal third swollen, ventral view: absent = 0; present = 1.
92. Abdomen, physogastry: absent = 0; present = 1.
93. Male genitalia, parameres: more or less symmetrical = 0; strongly asymmetrical = 1.
94. Male genitalia, parameres, basal apophyses: absent = 0; present = 1.
95. Female genitalia, styli: absent = 0; present = 1.
96. Female genitalia, vaginal palpi: rounded, about as long as wide = 0; elongate, at least twice as long as wide = 1.
97. Female genitalia, bursa copulatrix, sclerites: absent = 0; present = 1.
98. Female genitalia, bursa copulatrix, shape of sclerites: plate-like = 0; spicule-like = 1; ring-like = 2.

Supplementary Note 2. Additional remarks of central hypothesis, methods and discussion of the defensive strength analyses

Central hypothesis. In the case of being attacked by predators or falling from a deadly height, physical defence is important for insects. For Ceratocanthinae, a few studies hypothesized that conglobation behaviours have defensive functions. Here, we try to quantify the defensive strength of scarab beetles, especially Ceratocanthinae, and analyse how many factors contributed to this feature by employing an engineering approach.

The central hypothesis that **defensive strength was mainly determined by the shape, dimensions (material thickness), and material mechanical properties of the exoskeleton** (Fig. 3a) is derived from the principles of engineering mechanics[1]. Generally, the ability of a structure that withstands loading (a bridge, for instance) to resist deformation is not only positively proportional to its dimensions and material properties but also highly affected by its geometry (for example, triangular structures are stronger in terms of bearing shears than rectangular structures). Similar biomechanical phenomena have also been observed in animals' skeletal systems since their main functions include load bearing[2]. Bones tend to grow thicker to bear extra loads. For example, the upper-extremity bones of professional tennis players have developed a profoundly thicker cortical thickness (approximately 30%) on their playing side than on the control side to improve mechanical strength to withstand more impacts[2]. In another example, although fractured bone can self-repair (if the crack is small enough), the material properties of neo-bone are not as strong as those of healthy bone[3]. To compensate for this material property deficiency, the repaired bone normally grows thicker in diameter. We applied these principles and previous knowledge to understand how beetles balance the shape, thickness (dimensions), and material properties of the exoskeleton to achieve physical strength.

While it is difficult to measure the defensive strength of beetles. For simplify the problem, we **simulated the common defensive scenario**. For scarab beetles, most of the attackers are birds and mammals which body size much greater than that of beetles. And for pill scarab beetles, most of the attackers are solider ants or termites, which the length of mandibles equal or larger than the body size of the beetles. Therefore, we estimated the defensive strength of these exoskeletons by performing uniaxial compression tests on the whole bodies of beetles (Fig. 3B).

Methods and Results. As the specimens were compressed, the measured forces increased with intermittent drops, where we assumed that the exoskeleton fractured or that the connections between exoskeletal parts became disconnected. Although there were intraspecific variations between specimens for all species, the rolled Ceratocanthinae obviously exhibited the highest resistance to deformation in terms of the slope trend of curves and the force withstood by the samples before the first large fracture occurred (Fig. 3b). To quantitatively compare defensive strength between these species, the measured force at 10% relative deformation of the whole body height or at the first fracture (whichever occurred first) was recorded. At 10% relative deformation, rolled status Ceratocanthinae bore the greatest compressive force (3.76-4.82 N) without fracture (Fig. 3b), while open status bore Ceratocanthinae 0.34-4.08 N, Hybosorinae, 1.52-3.60 N, and Melolonthinae, 0.7-1.19 N. To reduce the effect of body size, the force was divided by the projected area of the beetles to represent stress. Hence, **the overall defensive strength of Ceratocanthinae was considered to be obviously greater** (p<0.001) than that of Hybosorinae and Melolonthinae. This normalized method penalized Ceratocanthinae with conglobation behaviours, in which the actual contact area should be less than the projected area and the real defensive strength should be higher than the result, but the difference was still sufficient to display the general comparative result.

Firstly, shape of exoskeleton was assumed to affect beetles' defensive ability based on both basic principles of mechanical engineering and research on stick insect *Carausius morosus*[4]. From uniaxial compression tests result (above) it is known that defensive strength of rolled status of Ceratocanthinae was obviously greater (p<0.001) than the open status which proved that **spherical body are benefit to the higher defensive stress** (Fig. 3b, x_1). For Ceratocanthinae, the advantages of spherical shape displayed also in some other aspects: protect the soft parts of the body and made itself difficult to carry by attackers.

Secondly, we measured the thickness of multiple cuticle parts of the whole body via micro-CT imaging. **The average thickness of the body walls (ratio of cuticle thickness to elytral length) of Ceratocanthinae was the highest** (p<0.05), and the variation in thickness among different parts of the body wall was also greater than that observed in Hybosorinae and Melolonthinae (Fig. 3c).

Thirdly, we analysed the material properties of the cuticle by nanoindentation tests. The elastic moduli (i.e., the unit force needed to deform a unit length) of outer exoskeleton parts (pronotum and

elytra) of Ceratocanthinae were profoundly higher than the four body walls of Hybosorinae and pronotum and elytra of Melolonthinae (Supplementary Table 3). These species showed a very large range of elastic moduli for different parts of the cuticle from 0.16 to 4.1 GPa, and the elytra of Ceratocanthinae possessed the highest elastic modulus (approximately 3.3 GPa on average). The different parts of cuticles have diverse mechanical properties, and their ranking differences among species may be associated with the ecological niches of the species (Fig. 3d). For Ceratocanthinae, the elastic moduli of the metasternum and abdominal ventrites were lower than those of the elytra and pronotum, while Melolonthinae showed the opposite results, which was maybe because the metasternum is inside the body in the Ceratocanthinae, where this body part has less opportunity to bear loads. Conversely, in Melolonthinae, the metasternum is exposed and bears various loads, especially when the insects fall from tress. Therefore, we suggest that the **species with stronger body walls are the ones subjected to greater environmental stress**.

Summary. In summary, the Ceartocanthinae with relatively high defensive strength in specific scenario among species of scarab beetles, owing to not only the spherical body shape but also the thickness and stronger mechanical properties of the dorsal cuticle.

Limitations. Here, we attempted to quantify the defensive strength of scarab beetles and explain how different factors contributed to them by applying an engineering approach. While there were systematic biases and/or simplifications in the quantification approaches—uniaxial compression, micro-CT, and nanoindentation for the evaluations of defence, thickness, and material properties, respectively—they were negligible for drawing qualitative conclusions. First, uniaxial compression is indeed a simplified evaluation of the physical defence of beetles in several ways. Measurements obtained from dead samples cannot truly represent how much deformation these beetles can withstand in nature. However, it is too difficult to test live specimens for such rare species. Hence, we used the measured forces at 10% deformation to represent the injury/mortality point in an attempt to represent the strength of the exoskeleton and used normalized height deformation and divided the projected area to offset the size variation between species. These beetles may be able to bear more than 10% deformation (when alive), but the force data dropped frequently after 10% for most of the samples, so it was difficult to identify an injury/mortality point (Fig. 3b). Notably, if the force instantly dropped by more than 5% of the maximum value during the compression test, we assumed

that there was a fracture and considered this the injury/mortality point (even at less than 10% deformation). Additionally, the impact speed and impact angle were neglected in our study due to the limited availability of samples (a constant speed of 250 μm/min was used).

Second, materials yield at the weakest point, normally at the thinnest point or the point with the greatest stress concentration, but the average thickness of the pronotum and elytra was used to compare groups. It would be more accurate to map the thickness of and stress on the exoskeleton by using FEA, therefore the FEA analysis were used in simulate ancestral elytra. Third, nanoindentation can only measure the material properties at a nanometre-scale depth, but it was used to represent the total thickness (at a micrometre scale) of the exoskeleton parts. We believe this was reasonable because the outermost layer on which the nanoindentation was conducted was the stiffest layer[5–7]. Hence, this measured layer and the nanometre depth of adjacent structures contributes the most to deformation resistance.

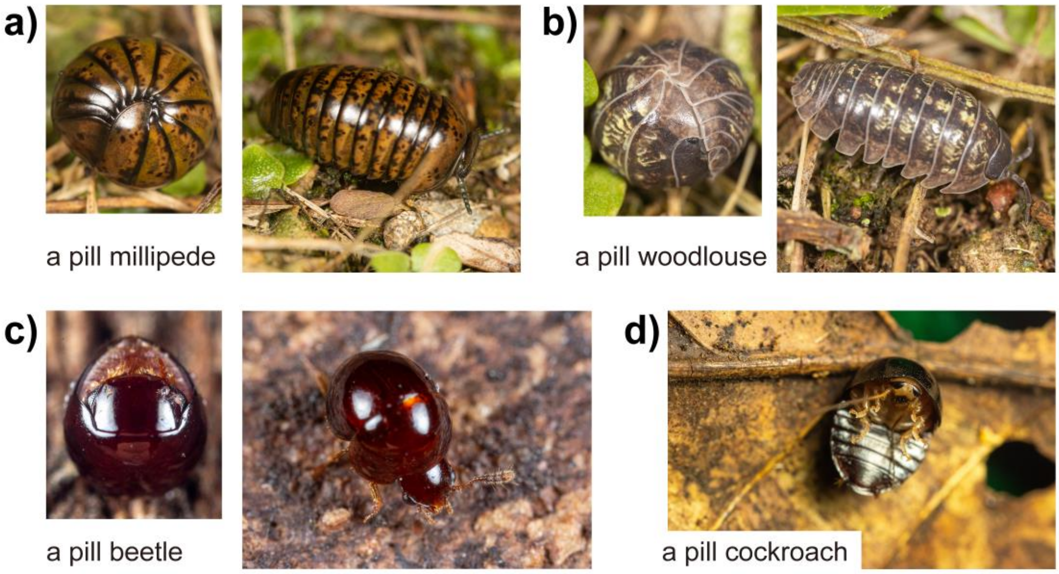

Supplementary Fig. 1. Examples of arthropods with conglobation behaviours. a) Complete conglobation—a pill millipede: *Procyliosoma* sp., New Zealand (Diplopoda: Sphaerotheriida: Procyliosomatidae). **b)** Complete conglobation—a pill woodlouse: *Armadillidium vulgare*, New Zealand (Crustacea: Isopoda: Armadillidiidae). **c)** Incomplete conglobation—a pill beetle: *Agathidium* sp., China (Insecta: Coleoptera: Leiodidae). **d)** Incomplete conglobation—a pill cockroach: *Perisphaerus* sp., China (Insecta: Blattaria: Blaberidae). ©Yandong Chen

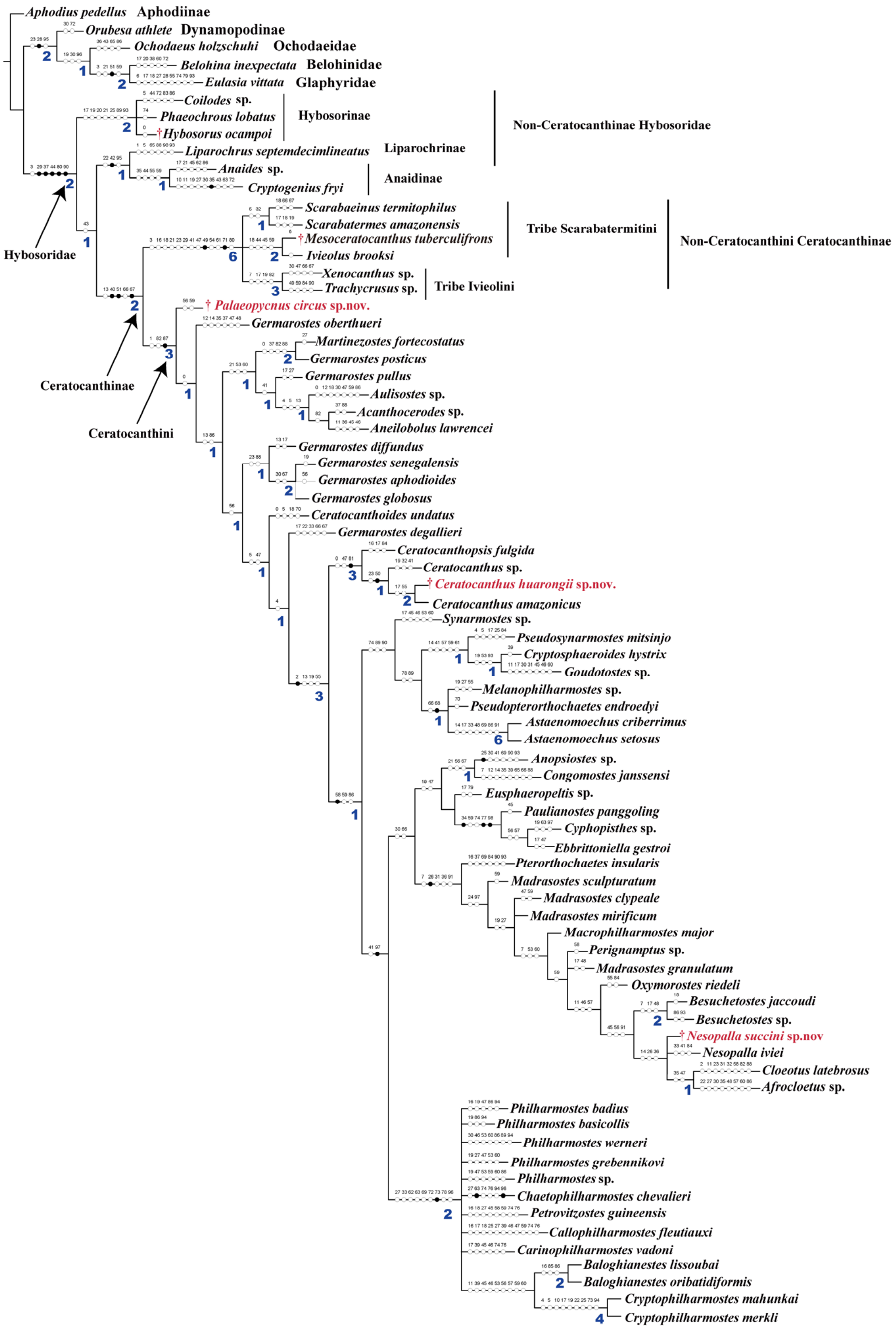

Supplementary Fig. 2. Strict consensus tree (tree length=564 steps, CI=0.21, RI=0.67) with bremer support in main node (blue numbers).

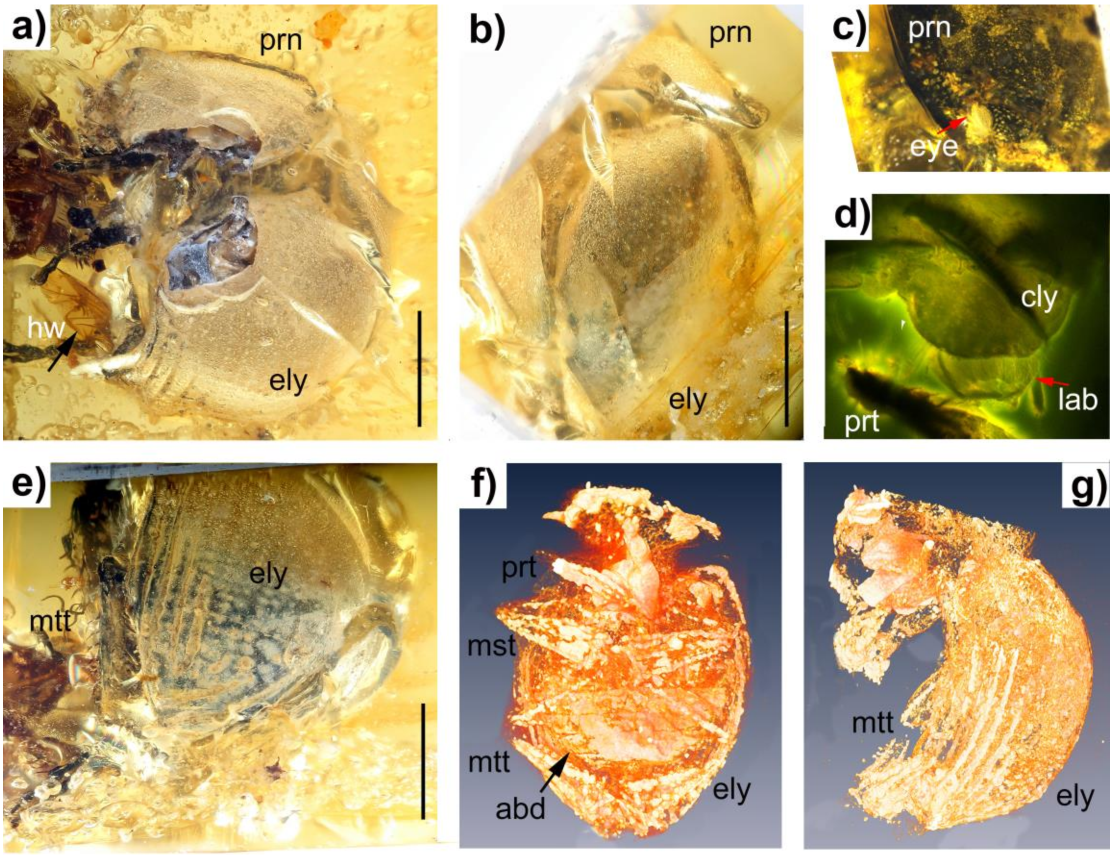

Supplementary Fig. 3. Material of *Palaeopycnus circus* Lu, Ballerio & Bai sp. nov. a) General habitus, lateral view, with the black arrow pointing to the hindwing. **b)** General habitus, dorsal view. **c)** Head, showing the clypeus and eyes. **d)** Head, showing the clypeus and labrum, under green epifluorescence. **e)** General habitus, lateral view. **f)** General habitus, ventral view. **g)** General habitus, lateral view. b), f)-g): Screenshot images from the 3D surface model. Scale bars: 1 mm. Abbreviations: prn, pronotum; ely, elytron; hw, hind wing; cly, clypeus; prt, protibia; lab, labrum; mst, mesotibia; mtt, metatibia; abd, abdomen.

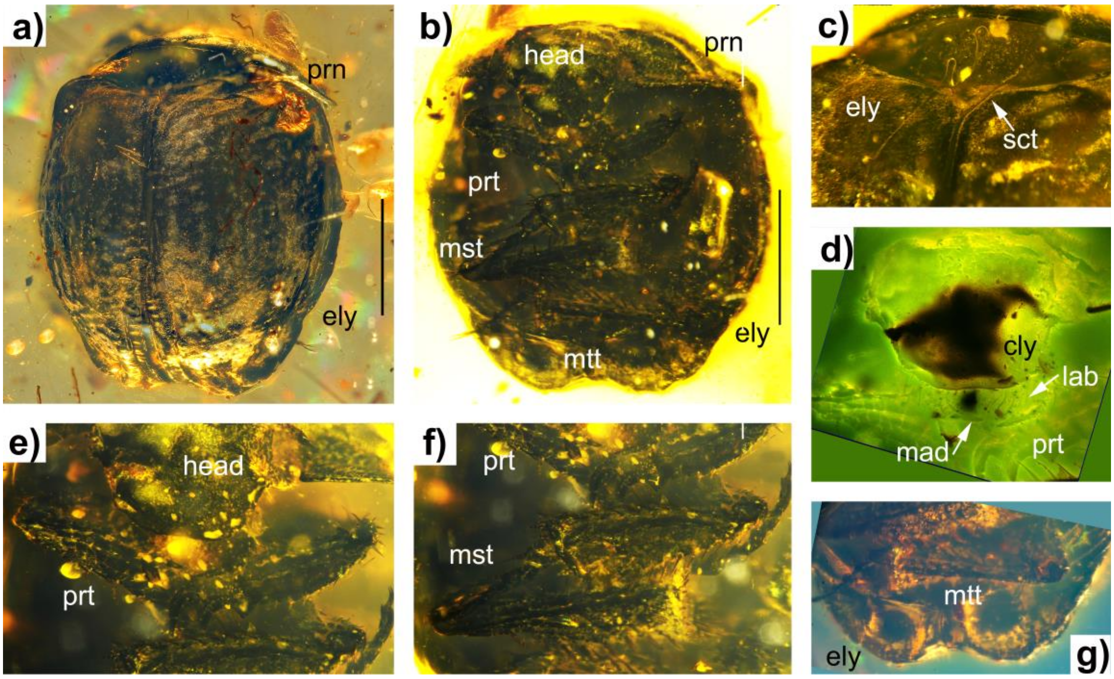

Supplementary Fig. 4. Photographs of *Palaeopycnus fushengii* Lu, Ballerio & Bai sp. nov. (holotype). **a)** General habitus, dorsal view. **b)** General habitus, ventral view. **c)** Elytra and scutellum, ventral view. **d)** Head, showing the clypeus and labrum, under green epifluorescence. **e)** Head and protibia, ventral view. **f)** Protibia and mesotibia, ventral view. **g)** Metatibia and the end of the elytra, ventral view. Scale bars: 1 mm. Abbreviations: prn, pronotum; ely, elytron; sct, scutellum; prt, protibia; cly, clypeus; lab, labrum; mad, mandible; mst, mesotibia; mtt, metatibia.

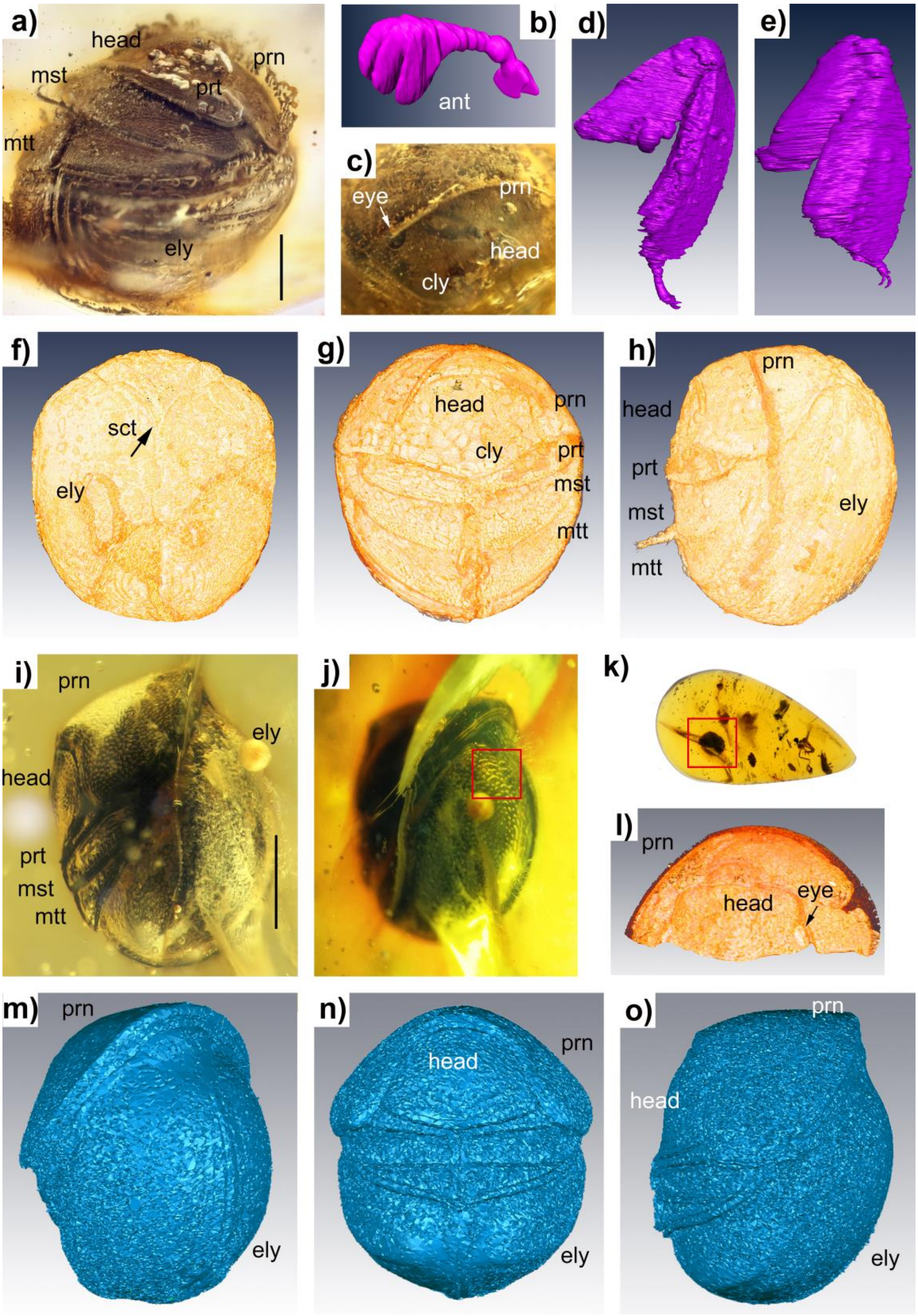

Supplementary Fig. 5. Images of *Ceratocanthus huarongii* Lu, Ballerio & Bai sp. nov. and *Nesopalla succini* Lu, Ballerio & Bai sp. nov. a)-h) *Ceratocanthus huarongii* Lu, Ballerio & Bai sp. nov. (holotype). **a)** General habitus, lateral view. **b)** Antenna. **c)** Head, showing the clypeus and eye. **d)** The left mesotibia. **e)** The left metatibia. **f)** General habitus, dorsal view. **g)** General habitus, ventral view. **h)**

General habitus, lateral view. **i)-o)** *Nesopalla succini* Lu, Ballerio & Bai sp. nov. (holotype). **i)** General habitus, lateral view. **j)** Overview of the amber. **k)** Overview of the amber. **l)** Lateral view, where the red rectangle and arrows show prosternal apophyses that reach the inner wall of the pronotum. **m)** General habitus, lateral dorsal view. **n)** General habitus, ventral view. **o)** General habitus, lateral view. a), c), i), j) Photographs. b), d)-h), m)-o) Screenshot images from the 3D surface model. k) Still image generated from shaded surface display volume rendering. Scale bars: 1 mm. Abbreviations: prn, pronotum; ely, elytron; prt, protibia; mst, mesotibia; mtt, metatibia.

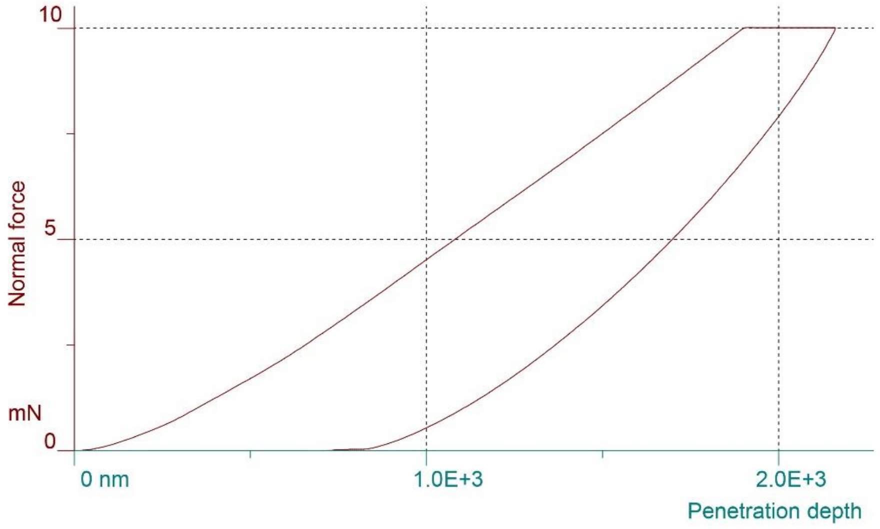

Supplementary Fig. 6. Load-displacement curve of nanoindentation test in pronotum of Ceratocanthinae (E*=1398.5 MPa).

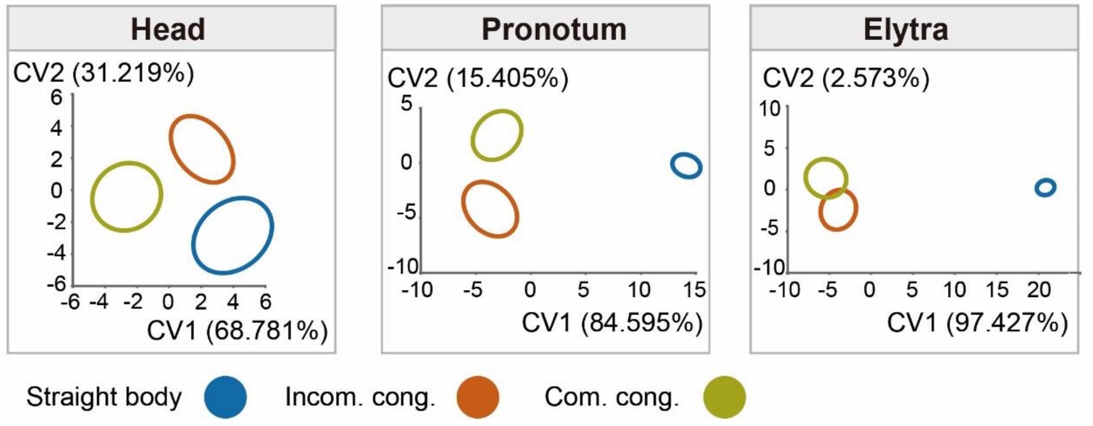

Supplementary Fig. 7. Results of canonical variate analysis (CVA) of basic shapes (head, pronotum and elytra).

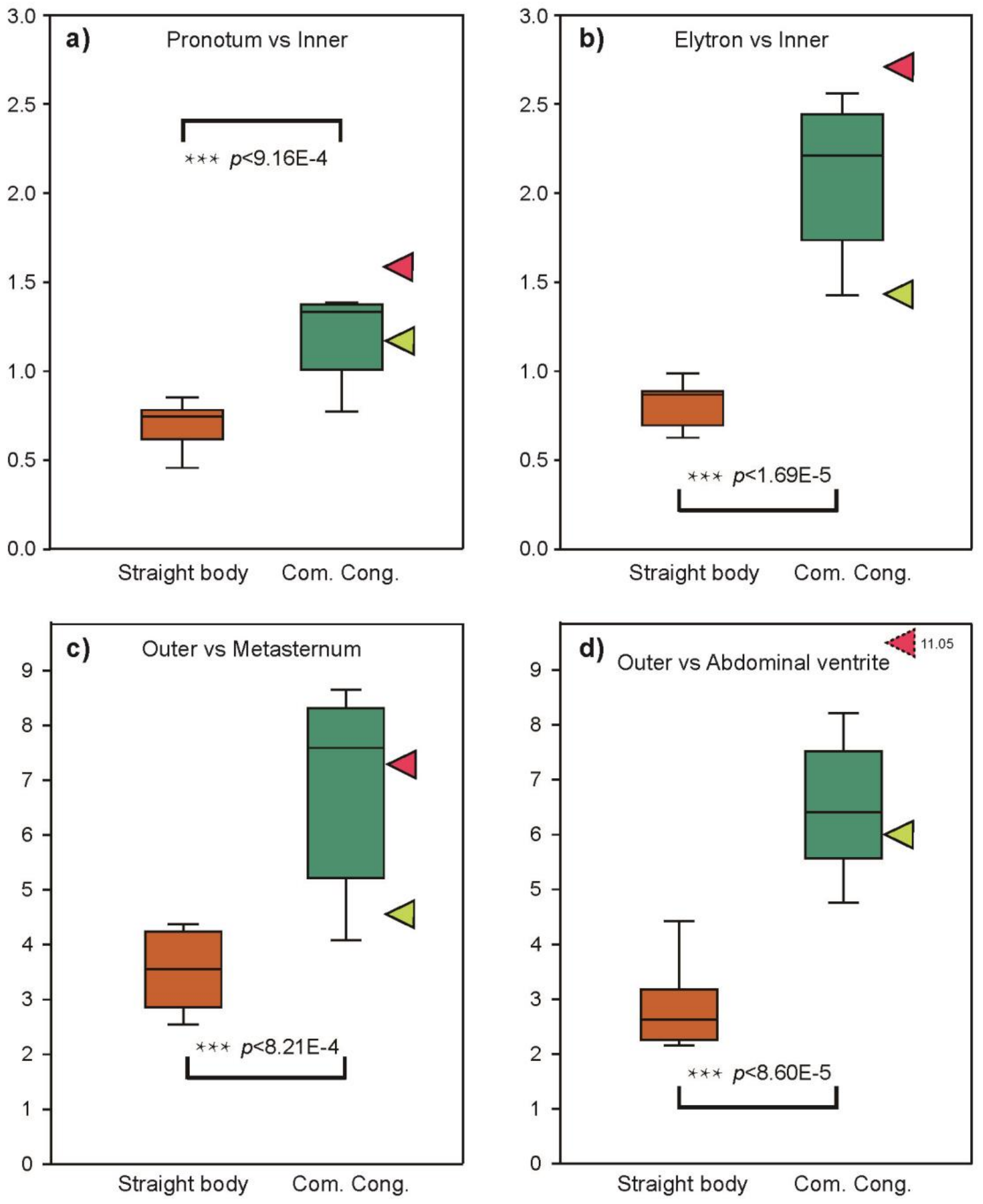

Supplementary Fig. 8. Morphometric analyses of the thickness of the body wall between species of scarab beetles with complete conglobation or straight bodies (see Supplementary Table 2)**. a)** Box plot showing the ratio of pronotum to inner surface (abdominal ventrites plus metasternum) thickness. **b)** Box plot showing the ratio of elytron to inner surface (abdominal ventrites plus metasternum) thickness. **c)** Box plot showing the ratio of outer surface (elytron plus pronotum) to metasternum thickness. **d)** Box plot showing the ratio of outer surface (elytron plus pronotum) to abdominal ventrite thickness. Green − yellow triangle: *Palaeopycnus circus* from Mesozoic amber, red triangle: *Nesopalla succini* from Cenozoic amber. ***: $p < 0.001$.

Supplementary Table 1. Diversity and archetypes of Ceratocanthinae that have spherical ability

Archetypes	Straight body	Incomplete conglobation	Complete conglobation
Extant species	Ivieolini (monogeneric) Scarabatermitini (Four genera)	Ceratocanthini: *Acanthocerodes*, *Aneilobolus*, *Aulisostes*, *Ceratocanthoides*, *Cloeotus*, *Germarostes*, *Martinezostes*	Ceratocanthini: The rest 31 genera
Extinct species	**Mesozoic:** *Mesoceratocanthus tuberculifrons*	**Cenozoic:** *Germarostes emarginatus* **(**Dominican amber**)**	Without
Extinct species (new)	/	**Mesozoic** (Kachin amber)	**Cenozoic** (Dominican amber)
Characters	-	head and pronotum markedly deflexed, the ventral partly exposed	elytra, pronotum, head, and all six tibiae forming a tightly connected external surface

Supplementary Table 2. The thickness of cuticle exoskeleton in Scarab beetles.

Archetype	Subfamily	Species	Length (mm)	Width (mm)	Thickness (mm)			
					Pronotum	Elytron	Abdominal ventrites	Metasternum
Com. cong.	Ceratocanthinae	*Paulianostes panggoling*	3.7637	3.1343	0.0457	0.0750	0.0177	0.0190
Com. cong.	Ceratocanthinae	*Eusphaeropeltis* sp. 1 Malaysia	3.2913	2.7337	0.0400	0.0663	0.0167	0.0133
Com. cong.	Ceratocanthinae	*Eusphaeropeltis* sp. 2 Malaysia	3.6790	3.3867	0.0363	0.0670	0.0217	0.0253
Com. cong.	Ceratocanthinae	*Pterorthochaetes* sp. 2 (China, Yunnan)	2.8433	2.3690	0.0703	0.1200	0.0297	0.0220
**Com. cong.**	**Ceratocanthinae**	***Pterorthochaetes* sp. 1 (Vietnam)**	**2.8127**	**1.7803**	**0.0693**	**0.1280**	**0.0240**	**0.0260**
**Straight body**	**Hybosorinae**	***Phaeochrous* sp.**	**8.4090**	**3.7920**	**0.0487**	**0.0563**	**0.0330**	**0.0240**
Straight body	Hybosorinae	*Hybosorus illigeri*	5.5923	2.4327	0.0430	0.0460	0.0300	0.0250
Straight body	Hybosorinae	*Hypseloderus* sp.	4.0147	1.7327	0.0463	0.0403	0.0340	0.0303
Straight body	Rutelinae	*Anomala aulax*	15.4257	7.6370	0.1123	0.1313	0.0550	0.0957
**Straight body**	**Melolonthinae**	***Apogonia cribricollis***	**9.3902**	**4.8772**	**0.0898**	**0.1032**	**0.0733**	**0.0455**
Straight body	Cetoniinae	*Cetonia aurata aurata*	18.7253	9.9130	0.1743	0.3383	0.2367	0.1443
Straight body	Aphodiinae	*Aphodius porcus*	2.8723	1.1180	0.0285	0.0320	0.0267	0.0193
Incomp. cong. (amber)	Ceratocanthinae	*Palaeopycnus circus* sp. nov.	2.2600	1.7900	0.0343	0.0425	0.0127	0.0177
Com. cong. (amber)	Ceratocanthinae	*Nesopalla succini* sp. nov.	3.2020	2.5790	0.0770	0.1330	0.0190	0.0293

Supplementary Table 3. Comparison of the elasticity moduli of body wall in scarab beetles. Significant differences are given for the elasticity moduli of the body wall in each species. ∗: $p < 0.05$, ∗∗: $p < 0.01$, and ∗∗∗: $p < 0.001$. CP: pronotum of Ceratocanthinae (n = 5), CE: elytra of Ceratocanthinae (n = 5), HP: pronotum of Hybosorinae (n = 5), HE: elytra of Hybosorinae (n = 5), HM: metasternum of Hybosorinae (n = 5), HA: abdominal ventrites of Hybosorinae (n = 5), MP: pronotum of Melolonthinae (n = 5), ME: elytra of Melolonthinae (n = 5), MM: metasternum of Melolonthinae (n = 5), MA: abdominal ventrites of Melolonthinae (n = 5).

	**CP**	**CE**	**HP**	**HE**	**HM**	**HA**	**MP**	**ME**	**MM**	**MA**
**CP**		***	***	***	***	***	***	***	**	**
**CE**			***	***	***	***	***	***	***	***

Supplementary Table 4. Species used in the analysis of geometric morphometric and ancestral characters' reconstruction.

Species	Head	Pronotum	Elytra
Cryptogenius fryi	/	*Anaides* sp.	*Anaides* sp.
Scarabaeinus termitophilus	/	*S. amazonensis*	*S. amazonensis*
Trachycrusus sp.	/	*Xenocanthus* sp.	*Xenocanthus* sp.
Mesoceratocanthus tuberculifrons	/	*Ivieolus brooksi*	*Ivieolus brooksi*
Hybosorus ocampoi	*Hybosorus illigeri*	/	/

Supplementary References

1. Gross, D., Hauger, W., Schröder, J., Wall, W. A. & Bonet, J. *Engineering mechanics 2 Mechanics of materials. 2nd Edition*. (Springer, 2018).
2. Jones, H. H., Priest, J. D., Hayes, W. C., Tichenor, C. C. & Nagel, D. A. Humeral hypertrophy in response to exercise. *J. Bone Jt. Surg.* **59**, 204–208 (1977).
3. Ekeland, A., Engesaeter, L. B. & Langeland, N. Mechanical-properties of fractured and intact rat femora evaluated by bending, torsional and tensile tests. *Acta Orthop. Scand.* **52**, 605–613 (1981).
4. Schmitt, M., Buescher, T. H., Gorb, S. N. & Rajabi, H. How does a slender tibia resist buckling? Effect of material, structural and geometric characteristics on buckling behaviour of the hindleg tibia in stick insect postembryonic development. *J. Exp. Biol.* **221**, jeb173047 (2018).
5. Peisker, H., Michels, J. & Gorb, S. N. Evidence for a material gradient in the adhesive tarsal setae of the ladybird beetle *Coccinella septempunctata*. *Nat. Commun.* **4**, 1–7 (2013).
6. Filippov, A. E., Matsumura, Y., Kovalev, A. E. & Gorb, S. N. Stiffness gradient of the beetle penis facilitates propulsion in the spiraled female spermathecal duct. *Sci. Rep.* **6**, 1–8 (2016).
7. Rajabi, H., Jafarpour, M., Darvizeh, A., Dirks, J. H. & Gorb, S. N. Stiffness distribution in insect cuticle: a continuous or a discontinuous profile? *J. R. Soc. Interface* **14**, 20170310 (2017).
