# Supplementary material for: The evolution of conglobation in Ceratocanthinae
Source: Commun Biol. 2022 Aug 6;5:777. doi: 10.1038/s42003-022-03685-2 (PMC9357020; doi:10.1038/s42003-022-03685-2)
Supplement: Supplementary file 2 — Description of Additional Supplementary Files [file 42003_2022_3685_MOESM2_ESM.pdf]

Description of Additional Supplementary Files

File name: Supplementary Data 1
Description: Character state matrix used in phylogenetic analysis.

File name: Supplementary Data 2
Description: Specimen permits.
