# Supplementary material for: The evolution of conglobation in Ceratocanthinae
Source: Commun Biol. 2022 Aug 6;5:777. doi: 10.1038/s42003-022-03685-2 (PMC9357020; doi:10.1038/s42003-022-03685-2)
Supplement: Supplementary file 3 — Supplementary Data 1 [file 42003_2022_3685_MOESM3_ESM.docx]

**Character state matrix used in phylogenetic analysis**

| Characters  Taxa | 00000000000000000000 00000000000000000000 00000000000000000000 00000000000000000000 00000000000000000  000000000011111111111 22222222223333333333 44444444445555555555 66666666667777777777 88888888889999999  01234567890123456789 01234567890123456789 01234567890123456789 01234567890123456789 01234567890123456 |
| --- | --- |
| *Palaeopycnus circus* (Kachin amber) | 010000001????00-????0?????????????0?????????10000002???11?01????????????01??????10101??10?1?0?????? |
| *Ceratocanthus huarongii* (Dominican amber) | 0110110011?1000-?0020???????????????????????10000012??0111?2????????????01???????010???10???0?????? |
| *Nesopalla succini* (Dominican amber) | 1110110011?0-011????????????????????????1???11120002??0000?0????????????01????????10???10???0?????? |
| *Hybosorus ocampoi* (fossil) | 100000000001010-??001???????????????????????10000001??010002???????????001??????10001??00?100?????? |
| *Mesoceratocanthus tuberculifrons* (fossil) | 000?0000???1000-?12100-?????????????????????010-0102??1??0?011??????????01??????00001?000??00?????? |
| *Aphodius pedellus* (Aphodiinae) | 00010200--00-10-10010101000000101----00-01010001000101110002120001110000010000100011100000200000-0- |
| *Belohina inexpectata* (Belohinidae) | 00000000-000-10-000010-0000210211----000000100000000??0100000----------011000000000110000020000???? |
| *Eulasia vittata* (Glaphyridae) | 000000100001010-022000-001000020100200100001000000000000000012100011000001100001000010000020010110- |
| *Orubesa athlete* (Dynamopodinae) | 000100000001010-010101000002100000020010000100000001010100021210001-000011000000000110000020000100- |
| *Ochodaeus holzschuhi* (Ochodaeidae) | 000100000000-10-011001000102102100011010010000000001010100021210011-000001000000000010100020000110- |
| *Coilodes* sp. | 000002000001010-020010-10102011010000111010100000001010100021210001-000011000000100110100210010000- |
| *Phaeochrous lobatus* | 000000000001010-020010-101020110100001100101100000010101000212100011000001100000100010000210010000- |
| *Anaides* sp. | 000000101001010-000100-100020110100101100110010000011100000012100011000001000000100010100010000100- |
| *Cryptogenius fryi* | 000000001010-10-0100012100010100100301100111000000011100000012010011000011000000100010000010000???- |
| *Liparochrus septemdecimlineatus* | 010002100001010-0101012100020110100001110110100000010101000212000111000001000000100010001000010100- |
| *Ivieolus brooksi* | 000100101001000-312100-0000000?0?0001???1000010-01021111000011--0000000101000000000010000020??????? |
| *Scarabaeinus termitophilus* | 000102001001000-312100-00002001000000???1000100101021111000211--00110001010000000000100000101?????? |
| *Scarabatermes amazonensis* | 000102001001000-300200-00002001000000???1000100101021111000211--00000001010000000000100000101?????? |
| *Trachycrusus* sp. | 000100011001000-121000-???0??01010000???100010010002??11000011--00000001010000000010000000201?????? |
| *Xenocanthus* sp. | 000100011001000-121000-00000002010000???1000100201021111000211--00110001010000000010100000101?????? |
| *Acanthocerodes* sp. | 110011001101000-010100-10002011010000011100010000002100101020----------001000010100011111010000000- |
| *Afrocloetus* sp. | 111011001100-0110101012100020101100201101000111010021000011010---------001000010101011110010000000- |
| *Aneilobolus lawrencei* | 110011001100-00-010100-10002011010001111100011100002100101020----------001000010100011110010000000- |
| *Anopsiostes* sp. | 111011001101100-010100-102020110100001101100100?00021100011112000011011001????101010?1010?0-010???? |
| *Astaenomoechus setosus* | 11101100110110100002010100020100110001101100100210021100011112000010111001110000101011110201000000- |
| *Astaenomoechus criberrimus* | 11101100110110100002010100020100110001101100100210021100011112000010111001110000101011110201000000- |
| *Aulisostes* sp. | 010011001101100-011100-10002012010000???100010020002100101000----------001000010101011010010000000- |
| *Baloghianestes oribatidiformis* | 111011001100-00-110201010001011011000111100011120002100000100----------0100000001010101100100000111 |
| *Baloghianestes lissoubai* | 111011001100-00-110201010001011011000111100011120002100000100----------0100000001010101100100000111 |
| *Besuchetostes* sp. | 111011011100-00-020101010011012110001110100011121002100000100----------001000010101011110010010000- |
| *Besuchetostes jaccoudi* | 111011011100-00-022101011011012110001110100011121002100000100----------001000010101011010010000000- |
| *Callophilharmostes fleutiauxi* | 111011001101100-30220101010201101100011110001011000211001110121100000100101010001010110100100000111 |
| *Carinophilharmostes vadoni* | 111011001101100-00020101000101101100011110001112000211001111121100000100101010001010110100100000111 |
| *Ceratocanthoides undatus* | 010002001101010-0111010100020120100001101100100200021101110212000000001001000010101011110010000000- |
| *Ceratocanthopsis fulgida* | 011011001101000-320201010002010010000110110010000002110011021200000000000100001011100?110010000000- |
| *Ceratocanthus amazonicus* | 011011001101000-0002010000020100100001101100100000121101110212000001000001000010111011110010000000- |
| *Ceratocanthus* sp. | 011011001101100-0101010000020100000001101000100000121100110212000000000001000010111011110010000000- |
| *Chaetophilharmostes chevalieri* | 111011001101100-01020101000201001100011010001002000211001111121200000100101010001010110100100010110 |
| *Cloeotus latebrosus* | 1100110011010011010101000001012000010110100011100002100000000----------0010000101000??011010000???? |
| *Congomostes janssensi* | 1110110111010011010100-100020120100201111000100000021100011112000101000001000010101011011010000???? |
| *Cryptophilharmostes mahunkai* | 111000001110-00-000101210101011011000111100011120002100000100----------0110000001010110100100010111 |
| *Cryptophilharmostes merkli* | 111000001110-00-000101210101011011000111100011120002100000100----------0110000001010110100100010111 |
| *Cryptosphaeroides hystrix* | 11101100110110110101010100020100100001111000100200021000101010---------001110000101011010200010000- |
| *Cyphopisthes* sp. | 111011001101100-0102010100020120101001101000100000021100001012010010001001100110101011010010000000- |
| *Ebbrittoniella gestroi* | 111011001101100-02010101000201201010011010001001000211000010120000100010011001101010110100100000013 |
| *Eusphaeropeltis* sp. | 111011001101100-02010100000201201000011010001000000211001111120000100010010000111010110100100000012 |
| *Germarostes aphodioides* | 110000001101010-0101010000020100100001101100100000021101010212000001000001000010101011111010000000- |
| *Germarostes degallieri* | 110011001101110-0001012100020120110001101100100200021101110212000011000001000010101011110010000000- |
| *Germarostes diffundus* | 110000001101000-0201010000020110100001101100100000021101110212000000000001000010101011111010000000- |
| *Germarostes globosus* | 110000001101010-0101010000020100100001101100100000021101110212000001000001000010101011111010000000- |
| *Germarostes oberthueri* | 11000000110110110101010100020110100200111100100210021101010212000000000001000010101011010010000000- |
| *Germarostes posticus* | 010000001101010-010100-10002011010000011110010000002100101020----------001000010100011111010000000- |
| *Germarostes pullus* | 110000001101010-000100-10001011010000111100010000002100101020----------001000010101011110010000000- |
| *Germarostes senegalensis* | 110000001101010-0102010000020100100001101100100000021101110212000001000001000010101011111010000000- |
| *Goudotostes* sp*.* | 111011001100-011020101010002012110000110100011120002100010100----------001110000101011010200010000- |
| *Macrophilharmostes major* | 111011001101100-010101011011012110001110100010020002100011110----------001000010101011010011000000- |
| *Madrasostes clypeale* | 111011011101100-0101010110110121100011101000100100021100111012000010000001000010101011010011000000- |
| *Madrasostes granulatum* | 111011001101100-020101011011012110001110100010021002100011100----------001000010101011010011000000- |
| *Madrasostes mirificum* | 111011011101100-0101010110110121100011101000100200021100111112000010000001000010101011010011000000- |
| *Madrasostes sculpturatum* | 111011011101100-0102010110120121100011101000100200021100111212000010000001000010101011010011000000- |
| *Martinezostes fortecostatus* | 010000001101010-010100-10001011010000011110010000002100101020----------001000010100011111010000000- |
| *Melanophilharmostes* sp. | 111011001101100-0101010100010100100001101100100200021101011112--1010101001110000101011010200000000- |
| *Nesopalla iviei* | 111011001100-011010101010001012111000110110011120002100000100----------00100001010100-010010000000- |
| *Oxymorostes riedeli* | 111011001100-00-010101011011012110001110100010120002100110100----------00100001010100-010011000000- |
| *Paulianostes panggoling* | 111011001101100-01010100000201201010011010001100000211001110120000100010011001101010110100100000013 |
| *Perignamptus* sp. | 111011001101100-010101011011012110001110100010020002100011000----------001000010101011010011000000- |
| *Petrovitzostes guineensis* | 111011001101100-3122010100020110110001101000110200021100110012-100000100101010001010110100100000111 |
| *Philharmostes* sp. | 111011001101100-010101010001010011000110100010010002100011100----------0100000001010111100100000111 |
| *Philharmostes badius* | 111011001101100-11010101000101001100011010001001000211001111121100000100100000001010111100100010111 |
| *Philharmostes basicollis* | 111011001101100-01010101000101001100011010001002000211001111121100000100100000001010111100100010111 |
| *Philharmostes grebennikovi* | 111011001101100-010101010002010011000110100010010002100011110----------0100000001010110100100000111 |
| *Philharmostes werneri* | 111011001101100-010201010001012011000110100010120002100011110----------0100000001010111101100010111 |
| *Pseudopterorthochaetes endroedyi* | 111011001101100-0102010100020100100001101100100200021100111112--1010100001110000101011010200000000- |
| *Pseudosynarmostes mitsinjo* | 11100000110110100002010101020100100001101000100200021100101011--000000100110000010100-010200000000- |
| *Pterorthochaetes insularis* | 111011011101100-110201010012012110001010100010020002110011111200001001000100001010100-0100010100012 |
| *Synarmostes* sp. | 111011001101100-020201010002010010000110110011120002100011110----------001100010101011010100000000- |
