# Supplementary material for: The evolution of conglobation in Ceratocanthinae
Source: Commun Biol. 2022 Aug 6;5:777. doi: 10.1038/s42003-022-03685-2 (PMC9357020; doi:10.1038/s42003-022-03685-2)
Supplement: Supplementary file 4 — Supplementary Data 2 [file 42003_2022_3685_MOESM4_ESM.pdf]

# Specimen Declaration

Here we declare the specimen provenance, specimen deposition and ethics oversight for the manuscript “The evolution of conglobation in Ceratocanthinae”. Five insect specimens from Burmese amber (three specimens) and Dominican amber (two specimens) are referred to in this study.

Three specimens of Burmese amber all collected before 2016. Specimens NIGP-Col-081 and NIGP-Col-053 are housed in the Nanjing Institute of Geology and Palaeontology, Chinese Academy of Sciences; specimens BXAM BA-COL-001 currently housed in the Institute of Zoology, Chinese Academy of Sciences (for ten years), will be eventually deposited in Beijing Xiachong Amber Museum. Details see the documents in Page 2-5.

Two specimens of Dominican amber (2016-H-Col-015 and 001493) both bought from China International Jewellery Fair. Details see the pictures of these Jewellery Fair which from the collectors (Page 6). Both of them housed in the Institute of Zoology, Chinese Academy of Sciences (No. 001493 for ten years), No. 001493 will be eventually deposited Three Gorges Entomological Museum, Chongqing, China. All specimens available for study by contacting MB. No. 001493 has illustrated in the book of Frozen Dimensions published 2017 but not formally described (See Pages 7-10).

Yours sincerely,

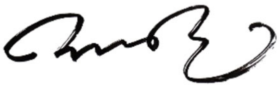

Ming BAI, Ph.D. Professor
Box 92, Institute of Zoology, Chinese Academy of Sciences
No. 1, Beichen West Road, Chaoyang District Beijing 100101, CHINA
Email: baim@ioz.ac.cn Tel: +86-10-64807928 Fax: +86-10-64807099

中国科学院南京地质古生物研究所
Nanjing Institute of Geology and Palaeontology, Chinese Academy of Sciences

# 标本说明函

本研究中涉及的两枚缅甸琥珀昆虫标本（标本收集号 NIGP-Col-081，NIGP-Col-053）为中国科学院南京地质古生物研究所（NIGP）永久馆藏标本。两枚标本于 2015 年收集。标本的收集和入库过程符合研究所化石标本采购条例规定。这两枚标本对其他研究者永久开放，免费研究。

中国科学院南京地质古生物研究所

2022 年 4 月 8 日

中国. 江苏. 南京市北京东路39号（邮编 210008） Add: 39 East Beijing Road, Nanjing 210008, P.R.China
Tel: 86-25-83282105; Fax: 86-25-83357026; E-mail: ngb@nigpas.ac.cn; Website: www.nigpas.cas.cn

中国科学院南京地质古生物研究所
Nanjing Institute of Geology and Palaeontology, Chinese Academy of Sciences

## Specimen Declaration

### 标本说明函

本研究中涉及的两枚缅甸琥珀昆虫标本（标本收集号 NIGP-Col-081，NIGP-Col-053）为中国科学院南京地质古生物研究所（NIGP）永久馆藏标本。两枚标本于 2015 年收集。标本的收集和入库过程符合研究所化石标本采购条例规定。这两枚标本对其他研究者永久开放，免费研究。

中国科学院南京地质古生物研究所
2022 年 4 月 8 日

Nanjing Institute of Geology and Palaeontology,
Chinese Academy of Sciences
8th April 2022

The two Burmese amber (No. NIGP-Col-081, NIGP-Col-053) which studied in this research were collected in 2015, and permanently housed in Nanjing Institute of Geology and Palaeontology, Chinese Academy of Sciences. The fossils were collected in full compliance with the regulations of our institute, and free open to the other researchers.

中国. 江苏. 南京市北京东路39号（邮编 210008） Add: 39 East Beijing Road, Nanjing 210008, P.R.China
Tel: 86-25-83282105; Fax: 86-25-83357026; E-mail: ngb@nigpas.ac.cn; Website: www.nigpas.cas.cn

**北京夏虫琥珀博物馆**

**Beijing Xiachong Amber Museum**

标本说明函

本研究中涉及的一枚缅甸琥珀昆虫标本（标本号 BXAM BA-COL-001）为北京夏虫博物馆（BXAM）于 2016 年以前采购的永久馆藏标本。该标本的收集和入库过程符合本馆化石采购条例规定，并对其他研究者永久开放，免费使用。

北京夏虫琥珀博物馆

2022 年 4 月 8 日

Dezhuo

Add: 9 Shuanghe Middle Road Beijing 100020, P.R.China
Tel: 0086 13552756822; E-mail: zhuode113@163.com

**北京夏虫琥珀博物馆**

**Beijing Xiachong Amber Museum**

标本说明函 **Specimen Declaration**

本研究中涉及的一枚缅甸琥珀昆虫标本（标本号 BXAM BA-COL-001）为北京夏虫博物馆（BXAM）于 2016 年以前采购的永久馆藏标本。该标本的收集和入库过程符合本馆化石采购条例规定，并对其他研究者永久开放，免费使用。

Beijing Xiachong Amber Museum
8th April 2022

北京夏虫琥珀博物馆

2022 年 4 月 8 日

Dezhuo

The one Burmese amber (No. BXAM BA-COL-001) which studied in this research belonging to Beijing Xiachong Amber Museum were collected before 2016. The specimen were collected of this specimens in full compliance with the regulations of our museum, and free open with other researchers.

Add: 9 Shuanghe Middle Road Beijing 100020, P.R.China
Tel: 0086 13552756822; E-mail: zhuode113@163.com

Some pictures in the China International Jewellery Fair

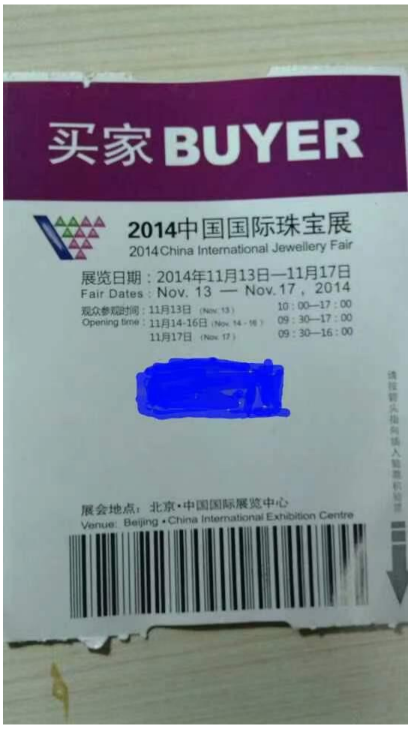


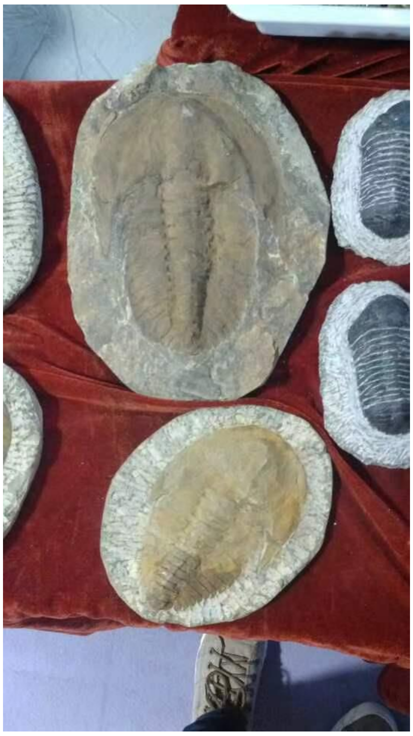

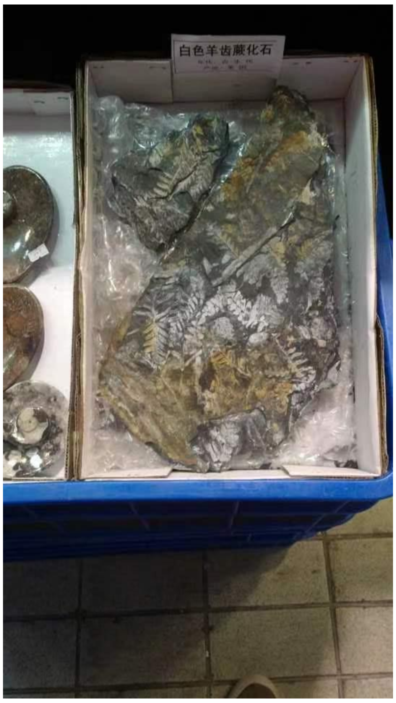


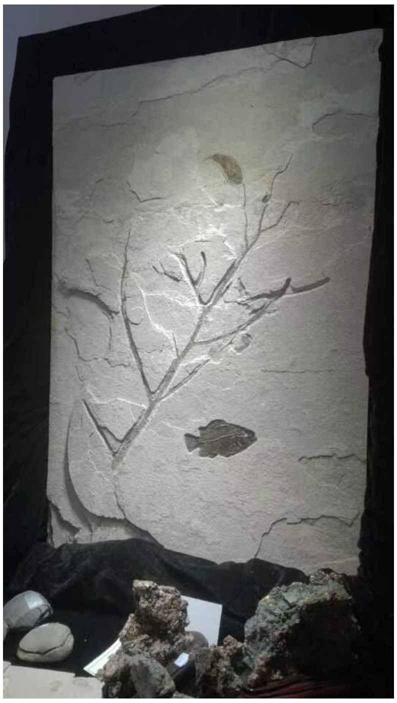

**三峡昆虫博物馆**

标本说明函

本研究中涉及的一枚多米尼加琥珀昆虫标本（标本号 001493）为三峡博物馆于 2014 年采购于北京国际珠宝展的永久馆藏标本，且该标本曾收录于 2017 年正式出版的《凝固的时空》第 431 页，后附相关页面和出版说明。该标本的收集和入库过程符合本馆化石采购条例规定，并对其他研究者永久开放，免费使用。

Specimen Declaration

The one Dominican amber (No. 001493) which studied in this research were collected from China International Jewellery Fair in 2014, and ever illustrated in the book Frozen Dimensions page 431， details see attachments. The fossil was collected in full compliance with the regulations of our institute, and free open to the other researchers.

三峡昆虫博物馆

Three Gorges Entomological Museum

[illegible]

2022.4.8

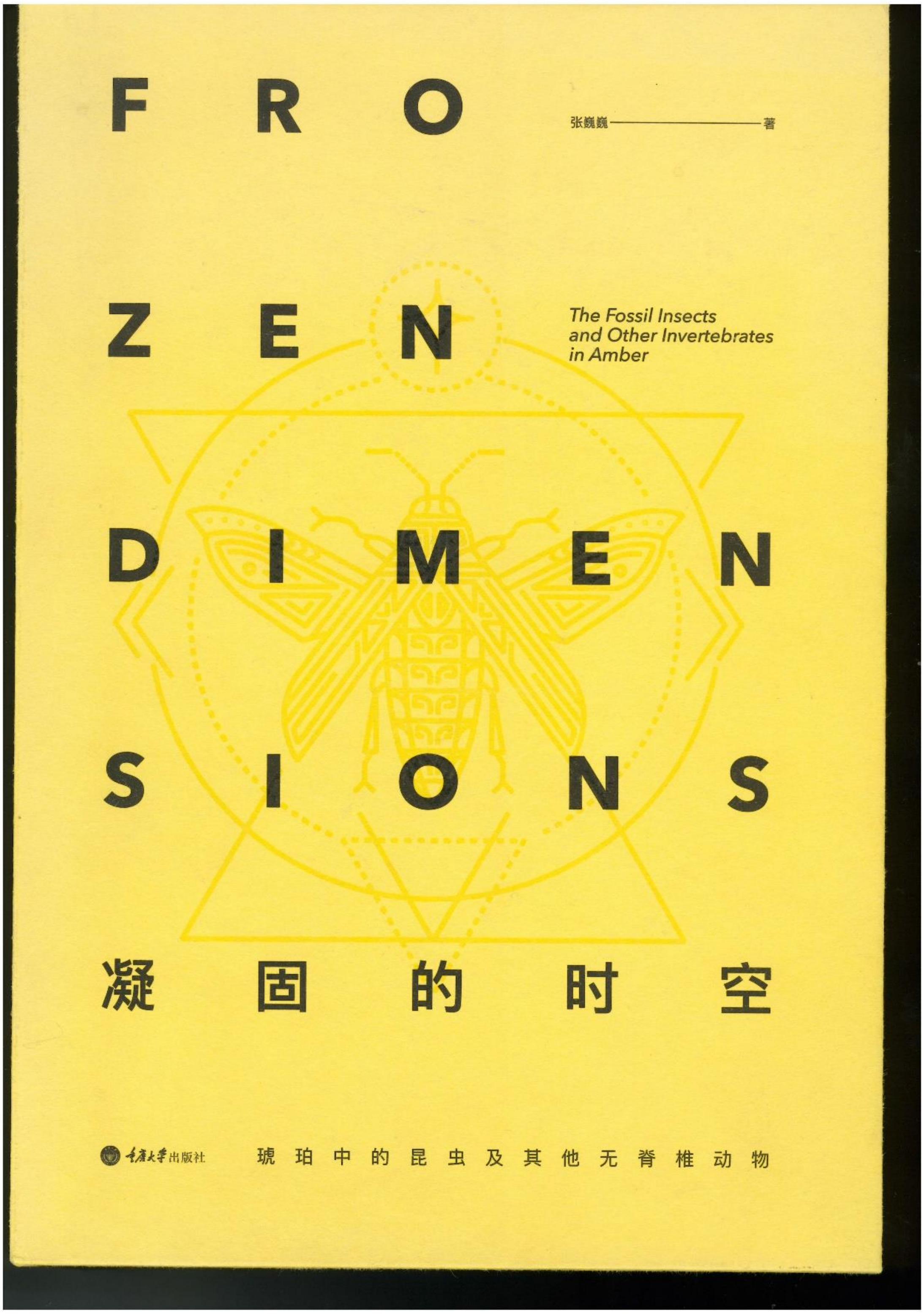
FRO
ZEN
DIMEN
SIONS
张巍巍——著
The Fossil Insects
and Other Invertebrates
in Amber
凝 固 的 时 空
重庆大学出版社
琥 珀 中 的 昆 虫 及 其 他 无 脊 椎 动 物

## 粪金龟科 *Geotrupidae*

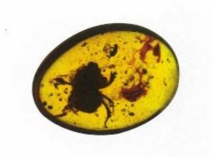

BU 粪金龟
Geotrupidae sp.

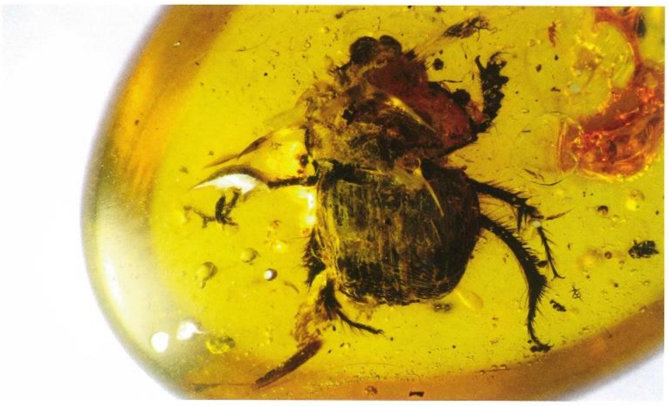

## g 驼金龟科 *Hybosoridae*

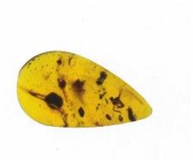

DO 球金龟
Ceratocanthinae sp.

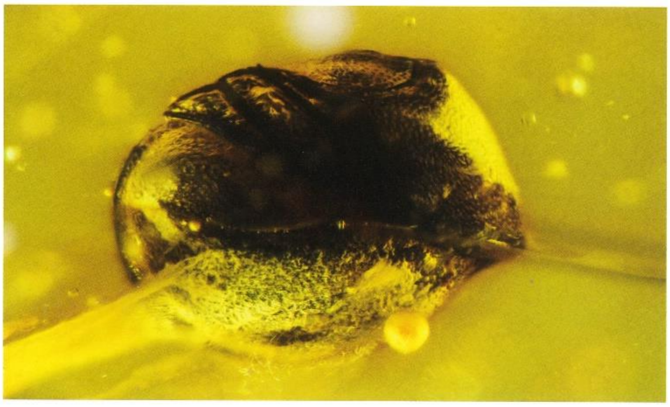

**内容提要**

虫珀是琥珀中最为奇特的品种，其中包裹了亿万年前的生命体，由于保存大多完好，甚至毫发无损，成为人们窥探远古世界的一扇窗口。

本书精选了产自缅甸、波罗的海和多米尼加的虫珀 800 件，向广大读者全面系统地介绍了琥珀中出现的无脊椎动物 6 门 12 纲 67 目的 6
了其他琥珀内含物（脊椎动物、植物、菌类等）的基本情况和世界各国的主要琥珀产地。

全书照片多达 2 000 余幅，是关于虫珀收藏和研究重要的文献资料。本书是古生物学家、昆虫爱好者及研究者、化石收藏爱好者、琥珀
备工具书，也可供广大生物学、地质学、珠宝学专业师生参考。

**图书在版编目（CIP）数据**

凝固的时空 琥珀中的昆虫及其他无脊椎动物 / 张巍巍著 . 一重庆： 重庆大学出版社，2017. 4
（好奇心书系）
ISBN 978-7-5624-9907-7

I. ①凝… II. ①张… III. ①琥珀一化石昆虫一研究 IV. ① Q915.81
中国版本图书馆 CIP 数据核字（2016）第 137252 号

**凝固的时空** 琥珀中的昆虫及其他无脊椎动物
**Ninggu de Shikong** Hupo zhong de Kunchong ji Qita Wujizhui Dongwu
张巍巍 著

**责任编辑：** 梁 涛
**责任校对：** 邹 忌
**装帧设计：** @broussaille 私制
**美术编辑：** 小 黑
**责任印制：** 张 策

**出版发行：** 重庆大学出版社
**出 版 人：** 易树平
**社　　址：** 重庆市沙坪坝区大学城西路 21 号
**邮　　编：** 401331
**电　　话：** 023-88617190 023-88617185（中小学）
**传　　真：** 023-88617186 023-88617166
**网　　址：** www.cqup.com.cn
**邮　　箱：** fxk@cqup.com.cn（营销中心）

**印　　刷：** 北京图文天地制版印刷有限公司
**开　　本：** 889mm × 1194mm 1/16
**印　　张：** 45.5
**字　　数：** 613 千字
**版　　次：** 2017 年 4 月第 1 版 2017 年 4 月第 1 次印刷
**书　　号：** ISBN 978-7-5624-9907-7
**定　　价：** 498.00 元

**全国新华书店经销**

本书如有印刷、装订等质量问题，本社负责调换
**版权所有，请勿擅自翻印和用本书制作各类出版物及配套用书，违者必究**
