# Supplementary material for: The evolution of conglobation in Ceratocanthinae
Source: Commun Biol. 2022 Aug 6;5:777. doi: 10.1038/s42003-022-03685-2 (PMC9357020; doi:10.1038/s42003-022-03685-2)
Supplement: Supplementary file 5 — Reporting Summary [file 42003_2022_3685_MOESM5_ESM.pdf]

nature portfolio

Corresponding author(s): Ming Bai

Last updated by author(s): Apr 14, 2022

Reporting Summary

Nature Portfolio wishes to improve the reproducibility of the work that we publish. This form provides structure for consistency and transparency in reporting. For further information on Nature Portfolio policies, see our Editorial Policies and the Editorial Policy Checklist.

Statistics

For all statistical analyses, confirm that the following items are present in the figure legend, table legend, main text, or Methods section.

n/a	Confirmed	
☐	☒	The exact sample size (n) for each experimental group/condition, given as a discrete number and unit of measurement
☐	☒	A statement on whether measurements were taken from distinct samples or whether the same sample was measured repeatedly
☐	☒	The statistical test(s) used AND whether they are one- or two-sided<br>*Only common tests should be described solely by name; describe more complex techniques in the Methods section.*
☒	☐	A description of all covariates tested
☒	☐	A description of any assumptions or corrections, such as tests of normality and adjustment for multiple comparisons
☒	☐	A full description of the statistical parameters including central tendency (e.g. means) or other basic estimates (e.g. regression coefficient) AND variation (e.g. standard deviation) or associated estimates of uncertainty (e.g. confidence intervals)
☐	☒	For null hypothesis testing, the test statistic (e.g. F, t, r) with confidence intervals, effect sizes, degrees of freedom and P value noted<br>*Give P values as exact values whenever suitable.*
☒	☐	For Bayesian analysis, information on the choice of priors and Markov chain Monte Carlo settings
☒	☐	For hierarchical and complex designs, identification of the appropriate level for tests and full reporting of outcomes
☒	☐	Estimates of effect sizes (e.g. Cohen's d, Pearson's r), indicating how they were calculated

Our web collection on statistics for biologists contains articles on many of the points above.

Software and code

Policy information about availability of computer code

Data collection	Zeiss Zen software (2012) were used to acquire images.
Data analysis	Amira 5.4, Geomagic Studio 2013, TNT version 1.5, WinClada, tps-DIG 2.05, MorphoJ 1.06a, Mesquite 3.61, 3Dmax 2021 and Z88Aurora V3 were used in this study (details see Methods)

For manuscripts utilizing custom algorithms or software that are central to the research but not yet described in published literature, software must be made available to editors and reviewers. We strongly encourage code deposition in a community repository (e.g. GitHub). See the Nature Portfolio guidelines for submitting code & software for further information.

Data

Policy information about availability of data

All manuscripts must include a data availability statement. This statement should provide the following information, where applicable:
- Accession codes, unique identifiers, or web links for publicly available datasets
- A description of any restrictions on data availability
- For clinical datasets or third party data, please ensure that the statement adheres to our policy

All raw and processed data will be made available upon request.

Field-specific reporting

Please select the one below that is the best fit for your research. If you are not sure, read the appropriate sections before making your selection.

☒ Life sciences ☐ Behavioural & social sciences ☐ Ecological, evolutionary & environmental sciences

For a reference copy of the document with all sections, see nature.com/documents/nr-reporting-summary-flat.pdf

Life sciences study design

All studies must disclose on these points even when the disclosure is negative.

Sample size	Sample size was firstly determined based on similar studies in this field.<br>For experiments involving quantification of defensive strength evaluation, material property analysis and thickness analysis, n=3 was chosen as the minimal replicate number, and sample size was determined by the number of available specimens within the replicates. We determined this to be sufficient owing to internal control and low observed variability among different groups.<br>For geometric morphometric analyses, n=14 was chosen as the minimal replicate number, and sample size was determined by the number of available species within the replicates. We determined this to be sufficient owing to internal control and low observed variability among different groups.
Data exclusions	Data were not excluded from analysis.
Replication	To ensure robust reproducibility: All measured data presented in this manuscript were repeated at least three times only a few exceptions (detalis see Methods). And all original specimens are still available for further replication.
Randomization	Id allocation was not random in this study because the status in different archetypes of insects are clearly defined and discrete.
Blinding	Blinding was not relevant to this study because this research need actively observing and describing the differences of characters.

Reporting for specific materials, systems and methods

We require information from authors about some types of materials, experimental systems and methods used in many studies. Here, indicate whether each material, system or method listed is relevant to your study. If you are not sure if a list item applies to your research, read the appropriate section before selecting a response.

Materials & experimental systems

n/a	Involved in the study	
☒	☐	Antibodies
☒	☐	Eukaryotic cell lines
☐	☒	Palaeontology and archaeology
☐	☒	Animals and other organisms
☒	☐	Human research participants
☒	☐	Clinical data
☐	☐	Dual use research of concern

Methods

n/a	Involved in the study	
☒	☐	ChIP-seq
☒	☐	Flow cytometry
☒	☐	MRI-based neuroimaging

Palaeontology and Archaeology

Specimen provenance	Five insect specimens from Burmese amber (three specimens) and Dominican amber (two specimens) are referred to in this study. Two Burmese amber are housed in the Nanjing Institute of Geology and Palaeontology, Chinese Academy of Sciences and one Burmese amber is housed in Beijing Xiachong Amber Museum. All three specimens collected before 2016 and in full compliance with the laws of related countries and China. Two Dominican amber will housed in the related museums and will deposited in Institute of Zoology, Chinese Academy of Sciences for ten years, and they are collected in full compliance with the laws of related countries and China.
Specimen deposition	The specimens have been deposited in the museums (details in the main text) and permit free access by other researchers.
Dating methods	No new dates are provided in this study.

☒ Tick this box to confirm that the raw and calibrated dates are available in the paper or in Supplementary Information.

Ethics oversight	No approval was required because only extinct specimens of insects used in this study and full compliance with the laws of related countries and China.

Note that full information on the approval of the study protocol must also be provided in the manuscript.

Animals and other organisms

Policy information about studies involving animals; ARRIVE guidelines recommended for reporting animal research

Laboratory animals	This study did not involve laboratory animals.
Wild animals	This study did not involve wild animals.
Field-collected samples	The dry pinned specimens of insects are deposited in the museum of Institute of Zoology, CSA and personal collections of Alberto Ballerio.
Ethics oversight	No approval was required because there are only dry specimens of insects used in this study.

Note that full information on the approval of the study protocol must also be provided in the manuscript.

Dual use research of concern

Policy information about dual use research of concern

Hazards

Could the accidental, deliberate or reckless misuse of agents or technologies generated in the work, or the application of information presented in the manuscript, pose a threat to:

No	Yes	
☒	☐	Public health
☒	☐	National security
☒	☐	Crops and/or livestock
☒	☐	Ecosystems
☒	☐	Any other significant area

Experiments of concern

Does the work involve any of these experiments of concern:

No	Yes	
☒	☐	Demonstrate how to render a vaccine ineffective
☒	☐	Confer resistance to therapeutically useful antibiotics or antiviral agents
☒	☐	Enhance the virulence of a pathogen or render a nonpathogen virulent
☒	☐	Increase transmissibility of a pathogen
☒	☐	Alter the host range of a pathogen
☒	☐	Enable evasion of diagnostic/detection modalities
☒	☐	Enable the weaponization of a biological agent or toxin
☒	☐	Any other potentially harmful combination of experiments and agents
